# Coffee Types and Type 2 Diabetes Mellitus: Large-Scale Cross-Phenotype Association Study and Mendelian Randomization Analysis

**DOI:** 10.3389/fendo.2022.818831

**Published:** 2022-02-11

**Authors:** Xinpei Wang, Jinzhu Jia, Tao Huang

**Affiliations:** ^1^ Department of Biostatistics, School of Public Health, Peking University, Beijing, China; ^2^ Center for Statistical Science, Peking University, Beijing, China; ^3^ Department of Epidemiology & Biostatistics, School of Public Health, Peking University, Beijing, China; ^4^ Department of Global Health, School of Public Health, Peking University, Beijing, China; ^5^ Key Laboratory of Molecular Cardiovascular Sciences (Peking University), Ministry of Education, Beijing, China

**Keywords:** type 2 diabetes mellitus, coffee types, coffee intake, cardiac metabolic risks, Mendelian randomization

## Abstract

**Purpose:**

To explore whether coffee intake is associated with the risk of type 2 diabetes mellitus (T2DM) from a genetic perspective, and whether this association remains the same among different types of coffee consumers.

**Methods:**

We utilized the summary-level results of 12 genome-wide association studies. First, we used linkage disequilibrium score regression and cross-phenotype association analysis to estimate the genetic correlation and identify shared genes between coffee intake and T2DM in addition to some other T2DM-related phenotypes. Second, we used Mendelian randomization (MR) analysis to test whether there is a significant genetically predicted causal association between coffee intake and the risk of T2DM or other T2DM-related phenotypes. For all the analyses above, we also conducted a separate analysis for different types of coffee consumers, in addition to total coffee intake.

**Results:**

Genetically, choice for ground coffee was significantly negatively associated with the risk of T2DM and some other related risks. While coffee intake and choice for decaffeinated/instant coffee had significant positive correlation with these risks. Between these genetically related phenotypes, there were 1571 genomic shared regions, of which 134 loci were novel. Enrichment analysis showed that these shared genes were significantly enriched in antigen processing related biological processes. MR analysis indicated that higher genetically proxied choice for ground coffee can reduce the risk of T2DM (T2DM: b: -0.2, p-value: 4.70×10^-10^; T2DM adjusted for body mass index (BMI): b: -0.11, p-value: 4.60×10^-5^), and BMI (b: -0.08, p-value: 6.50×10^-5^).

**Conclusions:**

Compared with other types of coffee, ground coffee has a significant negative genetic and genetically predicated causal relationship with the risk of T2DM. And this association is likely to be mediated by immunity. The effect of different coffee types on T2DM is not equal, researchers on coffee should pay more attention to distinguishing between coffee types.

## 1 Introduction

Coffee is one of the most widely consumed beverages in the world, with about 500 billion cups consumed yearly (1). Coffee contains a variety of chemical substances, although some of which have clear health effects, such as chlorogenic acids (CGAs), which can protect the body from oxidative damage (2), and ochratoxin A, which would aggravate obesity (3), there are still some coffee ingredients whose functions have not been clarified yet. And the content of components in coffee is also affected by various factors such as processing (4). Therefore, research on the effects of coffee types on human health despite being complicated is important.

In recent years, a plethora of studies have been published focusing on the association between coffee intake and type 2 diabetes mellitus (T2DM). Cumulative evidence from observational studies suggests a significant relationship between coffee intake and T2DM (5–7), but observational research is susceptible to confounding factors and reverse causality (8). This problem is somewhat overcome by randomized clinical trials (RCTs), but current RCTs on the impact of coffee intake on T2DM are faced with problems of short intervention period and inconsistent results (9–11). Some researchers used genetic variations as instrumental variables (IVs) to study the effect of genetic proxied coffee intake on T2DM by Mendelian randomization (MR), but these studies hardly distinguished coffee types and the results are not consistent with observational studies, or even completely opposite (12–14). Because of the inconsistent nature of findings regarding the impact of habitual coffee intake on T2DM, more research is necessary before health care workers can make evidence-based recommendations.

It has been indicated that people’s choices for coffee types vary (15) and there are differences in the chemical content of different coffee types (16–18). Previous research typically only investigated the association between total coffee consumption and T2DM risk without distinguishing among coffee types. Therefore, the results obtained from previous studies may not be applicable to every type of coffee, which may limit the clinical application thereof. Recently, the summary statistics of genome-wide association study (GWAS) on the total coffee intake and choices for different coffee types from the UK Biobank (UKB) large cohort have been released (19). Some of the loci found in the GWAS also have been reported to be associated with T2DM (19, 20), suggesting that there may be some genetic or causal links between them.

Therefore, the main objective of the present study is to explore whether coffee intake is associated with T2DM and some other T2DM-related phenotypes from a genetic perspective, and whether this association remains the same among different types of coffee consumers. Our study will shed light on the mechanism of the association between coffee consumption and T2DM and provide more precise guidance for coffee consumption.

## 2 Materials and Methods

### 2.1 Study Design

This study aims to identify the association of coffee intake/coffee types and T2DM from a genetic perspective. In order to explore the association more comprehensively, we also included some other T2DM-related phenotypes. The flowchart of the study is shown in **Supplementary Figure 1**. First, we calculated the genetic correlation between coffee intake/coffee types and T2DM with the use of linkage disequilibrium (LD) score regression. For the phenotypes with significant genetic correlation, we further conducted cross-phenotype association analysis to identify the shared genes and enrichment analysis to find out the enriched biological processes and pathways of these genes. Second, we used MR to estimate the effect of genetically proxied coffee intake/coffee types on T2DM and T2DM-related phenotypes. It is hoped that this study will lead to new insights of the association between coffee intake and T2DM.

In addition to total coffee intake, our research also included an analysis of the choice for different coffee types, including decaffeinated coffee (any type), instant coffee, ground coffee (include espresso, filter etc), and other types of coffee. In addition to T2DM, we also analyzed some other T2DM-related phenotypes that consists of body mass index (BMI), fasting glucose (FG), fasting insulin (FI), insulin resistance (HOMA-IR), and beta-cell function (HOMA-β).

### 2.2 Data Sources

The data used in this study were obtained from 12 large-scale GWASs (**Supplementary Table 1**). The data of coffee intake and different coffee type choices came from GWAS of more than 320,000 UKB participants, who were asked how many cups of coffee they drank per day and what type of coffee they usually consumed (decaffeinated coffee (any type), instant coffee, ground coffee (include espresso, filter etc), and other types of coffee) (21), released by Neale lab (19).

The T2DM (adjusted and unadjusted for BMI) data came from the largest T2DM GWAS meta-analysis, with a total of 898,130 participants (22). BMI data was obtained from a meta-analysis of ∼700,000 individuals (23). And the data of FG, FI, HOMA-IR, HOMA - β were obtained from the meta-analysis of 21 GWAS by Jos é e Dupuis et al. (24). For details of these studies, please refer to **Supplementary Table 1** and corresponding literature.

### 2.3 Statistical Analysis

#### 2.3.1 Linkage Disequilibrium Score Regression Analysis

As a convenient and frequently used tool in the study of genetic correlation between different phenotypes, LDSC relies on that the product of z-scores from two studies of phenotypes with non-zero genetic correlation is related to the LD score of the SNP under a polygenic model (25). This method requires only GWAS summary statistics and therefore is faster than other methods. We used LDSC to calculate the genetic correlation between coffee intake, choice for different coffee types and T2DM. We have adopted a series of quality control processes to ensure the accuracy of the results. To ensure the quality of SNP genotyping and imputation, only SNPs present in Hapmap3 were included in the analysis, and a variant was removed from the analysis if it had one of the following conditions: had missing values, INFO score <= 0.9, minor allele frequency (MAF) <= 0.01, p-value<0 or p-value>1, low sample size, not SNPs (e.g., indels), strand ambiguous SNPs, had duplicated rs numbers. Besides, we also checked that the median value of the signed summary statistic column was close to the null median in order to make sure that this column is not mislabeled. Bonferroni correction was performed on the obtained p-value and tests were judged statistically significant at p-value < 1.43×10^-3^ (0.05/5/7).

#### 2.3.2 Cross-Phenotype Association Analysis

Having identified the significantly genetically related phenotype-pairs, we further conducted cross-phenotype association (26) analysis to identify the shared genes of these phenotype pairs. This was implemented using the R software package ‘CPASSOC’ (26). This package uses the square root of the sample size of each phenotype as the weight and estimates the correlation matrix through summary statistics of all independent SNPs in the two phenotypes (26). This method not only considers the heterogeneity within the same phenotype or between different phenotypes, but also accounts for the potential kinship or population stratification between the participants (26).

SNPs with cross-phenotype association analysis p-value < 5×10^-8^ and single trait GWAS p-value < 0.05 were thought to have significant influence on both phenotypes. Then we used PLINK 1.9 software (27) to divide these SNPs into different independent clumping regions. SNPs with a distance less than 10,000kb and LD score R^2^ > 0.001 were divided into the same clumping region. And the SNP with the lowest p-value in each region was taken as the index SNP. A genomic region was judged as a novel shared genomic region if it meets the following four conditions at the same time: (1) was identified in the cross-phenotype association study (p-value < 5 × 10^-8^); (2) was not identified in the single phenotype GWAS (p-value > 5 × 10^-8^); (3) was not reported to be associated with either of the two phenotypes in the GWAS catalog (28); (4) was not reported to be associated with either of the two phenotypes in the Phenoscanner (29, 30).

#### 2.3.3 KEGG and GO Enrichment Analysis

In order to have a deeper understanding of the shared mechanism between coffee intake and T2DM, we performed Kyoto Encyclopedia of Genes and Genomes (KEGG) and Gene Ontology (GO) enrichment analysis on the shared genes identified in the previous step. All analysis is done through the R package ‘clusterProfiler’ (31).

#### 2.3.4 Mendelian Randomization Analysis

MR is currently a widely accepted method for assessing potential causal relationship for its unique advantages, compared with observational studies and RCTs (8). The core of MR is IV. In genetic epidemiology, IVs refer to genetic variations related to exposure but not directly related to outcomes and confounders. The selected IVs must satisfy three basic assumptions (8): first, it must be related to the exposure of interest; second, it must be independent of confounding factors; third, given exposure and confounders, the IVs are independent of the outcome.

We used two packages which are widely available to conduct MR: “TwoSampleMR” (32, 33) and “CAUSE” (34). “TwoSampleMR” is a convenient and frequently used tool in the study of two-sample MR. Using this package, we selected independent genetic variations with the threshold of p-value 5×10^-8^ and clump-kb 10000, clump-r2 0.01 as IVs, calculated causal estimates based on the association effect sizes of IVs with outcome and exposure, and then used a series of methods including inverse variance weighted method (IVW), MR-Egger regression, simple median, weighted median, penalized weighted median, simple mode, and weighted mode to obtain the overall causal estimate. We also tested whether the MR-Egger regression (33) intercept item was zero to check whether the horizontal pleiotropy was balanced.

Different from “TwoSampleMR”, as a recently published method, “CAUSE” uses all SNPs in estimating causal effects, not just variants that are strongly correlated with exposure (34). This method relies on that if exposure has a causal effect on the outcome, then for any SNP that has a non-zero effect on the exposure, its association effect size with exposure and outcome should be related. Based on this, this method is thought to be able to distinguish between causality and horizontal pleiotropy (related and unrelated). If the result shows that the causal model is better to fit the data than the shared model (p-value <0.05), then we think that exposure has a causal effect on the outcome.

## 3 Results

### 3.1 Genetic Correlation

**Figure 1:**
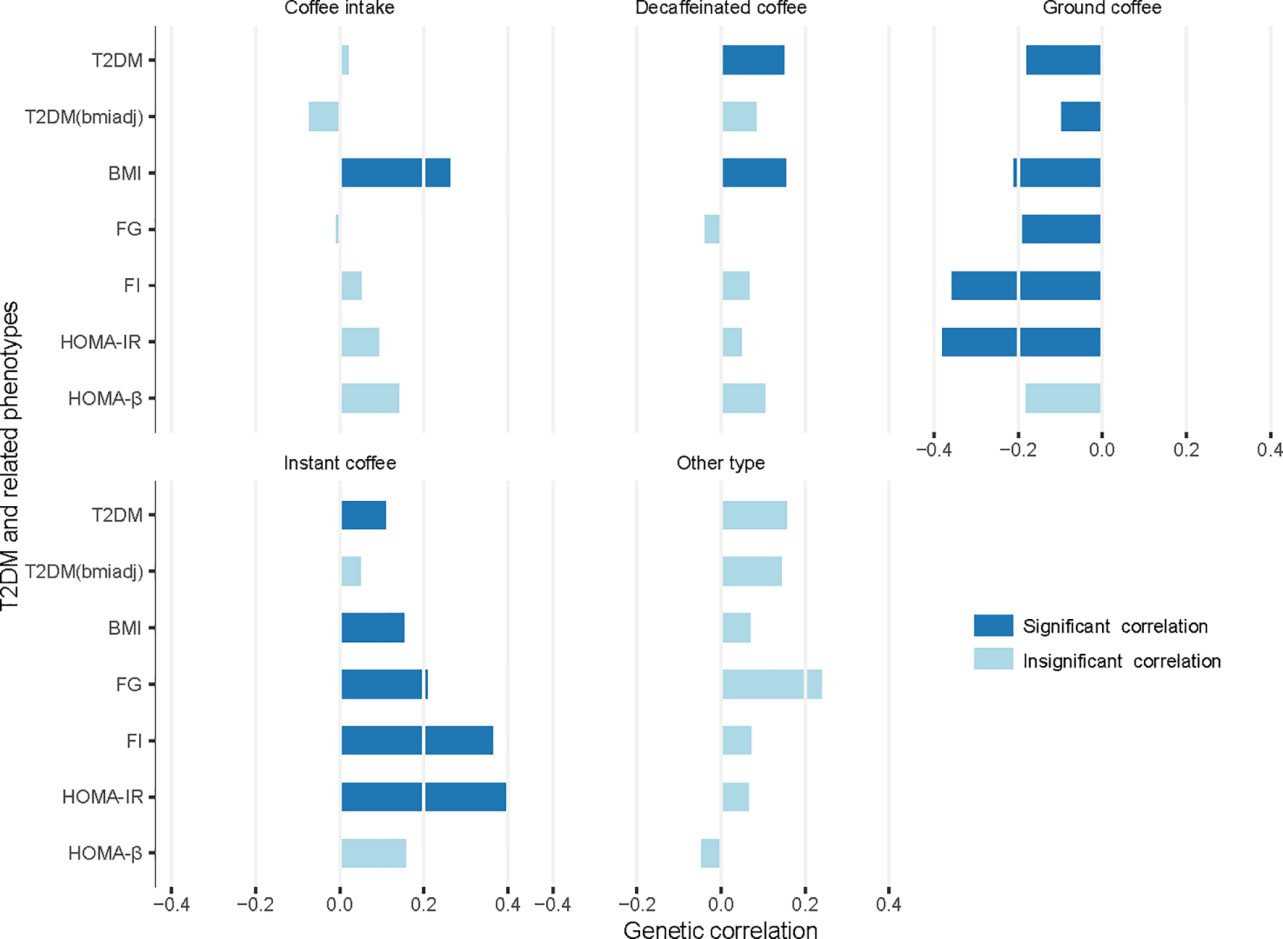
Genetic correlation between coffee intake or choices for different coffee types and T2DM/related phenotypes. T2DM, type 2 diabetes mellitus; T2DM(bmiadj), T2DM adjusted for body mass index (BMI); FG, fasting glucose; FI, fasting insulin; HOMA-IR, insulin resistance; HOMA-B, beta-cell function; Significant correlation, p-value < 1.43×10^-3^ (0.05/5/7).

There were extensive significant genetic correlations between the amount of coffee intake or choices for different coffee types and T2DM in addition to other T2DM-related phenotypes (**Figure 1**, **Supplementary Tables 2–6**). Without distinguishing between coffee types, we found that the amount of coffee intake was positively genetically associated with BMI (Rg = 0.2617, p-value = 1.12×10^-30^).

When considering different coffee types, there was a considerable difference between various coffee type choices. Choosing decaffeinated coffee had a significant positive genetic correlation with T2DM (Rg = 0.1496, p-value = 1.00×10^-4^) and BMI (Rg = 0.1541, p-value = 2.96×10^-6^), compared to other types of coffee. Choice for instant coffee showed more significant associations. It was positively associated with T2DM (Rg = 0.1090, p-value = 1.00×10^-3^), BMI (Rg = 0.1522, p-value = 1.73×10^-7^), FG (Rg = 0.2084, p-value = 1.00×10^-3^), FI (Rg = 0.3643, p-value = 6.40×10^-6^), and HOMA-IR (Rg = 0.3989, p-value = 4.86×10^-6^).

Conversely, choice for ground coffee showed a favorable relationship with all six phenotypes except HOMA-β (T2DM: Rg = -0.1805, p-value = 2.81×10^-13^; T2DM adjusted for BMI: Rg = -0.0979, p-value = 3.00×10^-4^; BMI: Rg = -0.2104, p-value = 6.40×10^-26^; FG: Rg = -0.1903, p-value = 9.39×10^-5^; FI: Rg = -0.3584, p-value = 6.88×10^-8^; HOMA-IR: Rg = -0.3809, p-value = 2.72×10^-8^). There was no significant genetic association between choice for coffee other than the above-mentioned types and T2DM or any related phenotype.

### 3.2 Cross-Phenotype Association Analysis

We identified multiple common genomic regions shared between the amount of coffee intake or choice for coffee types and T2DM or other T2DM-related phenotypes through cross-phenotype association analysis of trait pairs with significant genetic correlations (**Supplementary Tables 7–20**), including many novel regions which have not been reported in previous studies (**Tables 1A–D**).

Table 1ANovel shared genomic regions shared between coffee intake and T2DM or T2DM-related phenotypes.

**Table:** 

T2DM/related phenotypes	CHR	N	POS	KB	Index SNP	A1	A2	Coffee type		T2DM/related phenotypes		CPASSOC
								beta	p	beta	p	p
BMI	1	7	chr1:189946070-189961726	15.657	rs12046184	A	T	0.014	4.49E-04	-0.020	1.20E-06	2.93E-10
	1	19	chr1:99184056-99242884	58.829	rs1982703	G	A	0.005	6.02E-03	-0.009	2.90E-06	1.21E-08
	2	1	chr2:32920358-32920358	0.001	rs7564044	G	T	-0.008	2.21E-04	0.009	1.10E-04	3.75E-08
	2	1	chr2:49894154-49894154	0.001	rs10190188	C	T	-0.005	2.45E-03	0.009	8.70E-06	1.41E-08
	3	1	chr3:29088999-29088999	0.001	rs6796246	G	A	0.007	1.89E-05	0.008	1.30E-06	5.51E-09
	4	1	chr4:159851039-159851039	0.001	rs10031800	T	C	0.005	2.69E-03	-0.008	1.10E-05	3.71E-08
	4	1	chr4:173167398-173167398	0.001	rs836322	A	G	-0.005	8.46E-03	0.008	3.50E-06	4.95E-08
	4	1	chr4:28647811-28647811	0.001	rs10027492	T	A	0.005	6.90E-03	-0.008	1.40E-06	6.34E-09
	5	1	chr5:158488326-158488326	0.001	rs17718288	C	G	0.004	1.06E-02	-0.008	5.90E-06	3.97E-08
	8	1	chr8:118946541-118946541	0.001	rs10955841	A	G	0.005	3.61E-03	0.010	1.70E-07	1.64E-08
	8	1	chr8:78956658-78956658	0.001	rs2219968	A	G	-0.006	2.97E-04	-0.009	1.10E-06	3.87E-08
	9	1	chr9:73798371-73798371	0.001	rs1329767	A	C	-0.004	4.49E-02	0.010	1.40E-07	6.50E-09
	10	2	chr10:93644552-93790523	145.972	rs7917710	A	C	-0.006	1.56E-03	-0.010	3.50E-07	3.82E-08
	12	1	chr12:56895503-56895503	0.001	rs2657909	C	T	-0.006	7.81E-04	-0.010	3.90E-07	3.08E-08
	12	1	chr12:61854732-61854732	0.001	rs2009164	C	T	0.004	2.51E-02	-0.011	5.90E-08	1.36E-09
	14	1	chr14:30575613-30575613	0.001	rs6571334	G	C	0.008	6.72E-05	-0.009	2.50E-05	1.55E-09
	16	6	chr16:5850793-5854110	3.318	rs17792339	C	A	-0.007	7.59E-05	0.007	1.80E-05	1.17E-09
	17	2	chr17:41495423-41498187	2.765	rs11079338	G	A	0.006	1.15E-03	0.010	9.10E-08	9.23E-09
	18	1	chr18:22632927-22632927	0.001	rs9963409	T	C	-0.004	1.84E-02	0.009	9.80E-07	1.16E-08
	18	14	chr18:73124672-73159173	34.502	rs12165099	A	G	0.004	2.30E-02	-0.009	3.20E-07	4.50E-09
	22	6	chr22:38156183-38268922	112.74	rs2413485	T	C	0.005	3.23E-03	-0.010	7.00E-08	1.69E-10

**Table 1B:** Novel shared genomic regions shared between choice for decaffeinated coffee and T2DM or T2DM-related phenotypes.

T2DM/related phenotypes	CHR	N	POS	KB	Index SNP	A1	A2	Coffee type		T2DM/related phenotypes		CPASSOC
								beta	p	beta	p	p
T2DM	2	1	chr2:60588713-60588713	0.001	rs243016	T	A	-0.015	2.61E-02	-0.036	8.00E-08	1.22E-08
	3	1	chr3:136005792-136005792	0.001	rs7653249	G	C	0.016	3.26E-02	0.039	1.80E-07	2.68E-08
	3	24	chr3:94029191-94051397	22.207	rs4857339	T	C	0.015	1.96E-02	-0.034	1.80E-07	8.31E-09
	6	9	chr6:127798402-127817401	19	rs11154414	T	C	0.016	1.73E-02	0.034	2.20E-07	2.06E-08
	6	1	chr6:20725694-20725694	0.001	rs62397653	C	A	0.027	4.76E-02	-0.072	2.70E-07	4.95E-08
	7	2	chr7:102086552-102086605	0.054	rs77655131	T	C	-0.024	1.50E-02	0.053	5.40E-08	3.71E-09
	7	1	chr7:28222877-28222877	0.001	rs139048357	A	C	-0.063	2.65E-02	-0.160	4.60E-07	4.26E-08
	7	1	chr7:77044764-77044764	0.001	rs9641219	G	T	0.015	2.00E-02	-0.033	3.90E-07	3.37E-08
	8	3	chr8:110059269-110073120	13.852	rs6469227	G	T	-0.013	4.18E-02	-0.034	2.60E-07	4.13E-08
	8	23	chr8:116464988-116549176	84.189	rs12114740	C	T	0.014	3.39E-02	0.034	1.40E-07	2.17E-08
	10	1	chr10:12252217-12252217	0.001	rs2271804	A	G	-0.013	3.69E-02	-0.033	1.40E-07	3.57E-08
	10	1	chr10:99161831-99161831	0.001	rs7079477	G	C	-0.015	3.86E-02	0.038	1.30E-07	1.21E-08
	13	1	chr13:80591311-80591311	0.001	rs7328113	A	C	0.014	3.91E-02	-0.035	2.30E-07	2.59E-08
	14	1	chr14:74954059-74954059	0.001	rs7149930	T	C	-0.013	3.74E-02	-0.034	2.00E-07	2.35E-08
	20	3	chr20:20066701-20069826	3.126	rs73125628	T	C	-0.015	3.55E-02	-0.038	1.40E-07	1.79E-08
BMI	1	1	chr1:177394187-177394187	0.001	rs10913339	G	A	0.018	4.15E-02	0.013	6.30E-08	4.09E-08
	1	2	chr1:221715102-221766906	51.805	rs12079987	A	G	0.057	5.80E-03	-0.028	6.40E-07	1.47E-08
	2	3	chr2:241344240-241367465	23.226	rs10199929	T	A	-0.014	3.45E-02	-0.010	5.20E-08	3.39E-08
	8	4	chr8:50957454-51029371	71.918	rs1903311	G	A	-0.023	2.50E-03	-0.010	8.10E-08	1.46E-09
	9	1	chr9:28544375-28544375	0.001	rs10968649	G	T	-0.021	2.21E-02	0.013	2.20E-07	3.05E-08
	10	6	chr10:970426-1001215	30.79	rs2282419	C	G	-0.025	4.44E-03	0.013	9.20E-08	1.69E-09
	11	1	chr11:371265-371265	0.001	rs11246136	A	C	-0.022	3.48E-02	-0.016	6.90E-08	2.37E-08
	14	4	chr14:97236360-97255679	19.32	rs10149171	A	G	-0.039	4.25E-04	0.014	2.60E-06	7.04E-09
	14	2	chr14:99757151-99758235	1.085	rs2748805	T	C	0.025	2.29E-04	-0.009	2.50E-06	4.76E-09
	17	1	chr17:5005311-5005311	0.001	rs6502843	G	A	-0.022	7.62E-04	-0.008	6.90E-07	2.50E-08

**Table 1C:** Novel shared genomic regions shared between choice for ground coffee and T2DM or T2DM-related phenotypes.

T2DM/related phenotypes	CHR	N	POS	KB	Index SNP	A1	A2	Coffee type		T2DM/related phenotypes		CPASSOC
								beta	p	beta	p	p
T2DM	1	1	chr1:229594310-229594310	0.001	rs10916480	G	A	-0.023	1.45E-02	0.051	6.10E-08	8.17E-09
	1	2	chr1:26726127-26726967	0.841	rs113466616	A	G	-0.015	3.13E-02	0.035	1.80E-07	4.62E-08
	2	5	chr2:163639107-163649690	10.584	rs12614955	T	C	0.017	1.69E-02	0.035	9.70E-07	2.60E-08
	2	4	chr2:208910738-208921257	10.52	rs10932228	G	A	-0.017	7.71E-03	-0.033	3.30E-07	8.38E-09
	2	1	chr2:27541053-27541053	0.001	rs4665966	G	C	0.015	3.57E-02	0.042	6.00E-08	3.45E-09
	2	2	chr2:30878707-30879117	0.411	rs2602778	A	G	-0.018	8.21E-03	0.034	3.60E-07	2.84E-08
	3	3	chr3:170562564-170572896	10.333	rs12488260	T	G	-0.015	2.29E-02	-0.034	1.90E-07	8.69E-09
	3	1	chr3:186698711-186698711	0.001	rs6775869	C	T	-0.016	1.64E-02	-0.034	5.80E-07	1.63E-08
	5	1	chr5:44800917-44800917	0.001	rs10462082	C	A	0.015	2.02E-02	0.032	1.00E-06	3.05E-08
	5	1	chr5:86520965-86520965	0.001	rs13185500	A	T	-0.015	4.65E-02	0.038	1.30E-07	4.57E-08
	6	7	chr6:126072853-126097457	24.605	rs1977141	G	A	0.015	2.21E-02	0.033	3.60E-07	2.11E-08
	8	3	chr8:129579579-129592794	13.216	rs2395822	T	C	-0.017	1.46E-02	-0.034	6.30E-07	2.28E-08
	8	5	chr8:19780310-19813180	32.871	rs113023641	A	G	0.030	9.09E-04	-0.048	1.50E-07	5.32E-09
	8	1	chr8:41440849-41440849	0.001	rs183323983	C	A	-0.107	6.03E-04	-0.170	1.20E-06	1.06E-08
	8	1	chr8:41586822-41586822	0.001	rs7016707	T	G	-0.023	2.94E-02	0.053	2.80E-07	2.85E-08
	9	10	chr9:96970677-96994063	23.387	rs10821317	T	C	-0.014	3.77E-02	0.036	1.70E-07	3.54E-08
	10	6	chr10:13480317-13482073	1.757	rs4750356	G	A	0.016	1.81E-02	0.034	5.00E-07	1.78E-08
	11	3	chr11:2163932-2164990	1.059	rs7111447	T	A	-0.031	8.72E-03	0.067	1.60E-07	2.91E-08
	11	18	chr11:43635499-43696917	61.419	rs2862961	A	G	0.017	1.37E-02	-0.036	2.00E-07	1.72E-08
	12	4	chr12:26346146-26347517	1.372	rs2343869	G	A	-0.014	2.84E-02	-0.032	4.50E-07	2.63E-08
	14	3	chr14:104008159-104011429	3.271	rs12891360	T	C	0.030	1.78E-05	0.032	8.00E-06	4.53E-09
	16	1	chr16:1129010-1129010	0.001	rs4988483	A	C	0.036	1.00E-02	0.079	1.40E-06	2.39E-08
	16	1	chr16:69653696-69653696	0.001	rs39999	G	C	0.036	3.97E-05	-0.041	4.60E-06	4.38E-08
	17	1	chr17:76792179-76792179	0.001	rs1044486	A	G	-0.015	1.92E-02	-0.031	9.40E-07	4.61E-08
	20	1	chr20:31903533-31903533	0.001	rs721970	G	A	0.026	3.39E-02	0.069	2.00E-07	8.02E-09
T2DM (adjusted for BMI)	2	2	chr2:112878404-112880587	2.184	rs62157841	A	G	0.015	2.44E-02	0.039	5.00E-07	3.66E-08
	3	1	chr3:23252089-23252089	0.001	rs79433447	C	A	0.038	4.77E-02	0.110	1.40E-07	2.47E-08
	3	1	chr3:54876573-54876573	0.001	rs111494834	T	C	-0.036	3.33E-02	0.110	1.80E-07	6.70E-09
	9	1	chr9:139256766-139256766	0.001	rs3829109	A	G	-0.016	2.68E-02	-0.044	1.60E-07	2.07E-08
	10	2	chr10:64598621-64620042	21.422	rs911610	T	C	0.018	5.84E-03	-0.038	6.60E-07	3.55E-08
	11	1	chr11:65405600-65405600	0.001	rs2306363	T	G	0.017	3.57E-02	-0.049	1.80E-07	3.85E-08
	15	1	chr15:77847802-77847802	0.001	rs34075648	C	G	-0.014	4.07E-02	0.043	8.20E-08	1.67E-08
	16	1	chr16:1129010-1129010	0.001	rs4988483	A	C	0.036	1.00E-02	0.091	4.30E-07	1.86E-08
	18	5	chr18:46158145-46169673	11.529	rs299727	A	G	-0.023	1.91E-02	0.057	5.00E-07	2.83E-08
	22	1	chr22:50771629-50771629	0.001	rs149127232	C	A	0.025	2.87E-03	0.049	1.70E-06	1.84E-08
BMI	1	2	chr1:110686949-110687493	0.545	rs6682201	G	T	-0.017	6.82E-03	-0.008	3.60E-06	1.94E-08
	1	2	chr1:195316969-195322984	6.016	rs12731553	G	T	-0.017	1.45E-02	-0.009	1.40E-06	2.80E-08
	1	1	chr1:221057646-221057646	0.001	rs2738755	T	C	-0.015	2.68E-02	-0.009	2.10E-06	4.55E-08
	2	1	chr2:16617417-16617417	0.001	rs4281911	T	C	-0.014	3.27E-02	0.010	1.40E-07	4.47E-08
	3	7	chr3:141701527-141715543	14.017	rs3817176	T	C	0.035	7.59E-05	-0.012	3.50E-07	5.79E-09
	3	4	chr3:38625709-38647780	22.072	rs7374289	T	C	0.018	4.88E-03	0.009	1.80E-07	1.17E-09
	4	1	chr4:104978031-104978031	0.001	rs950882	C	T	0.018	1.13E-02	0.009	3.00E-06	4.60E-08
	4	1	chr4:180156210-180156210	0.001	rs17747559	A	G	0.017	9.83E-03	-0.009	3.80E-07	4.00E-08
	5	1	chr5:142892428-142892428	0.001	rs6876238	A	C	-0.014	2.79E-02	-0.009	6.80E-07	4.77E-08
	5	1	chr5:153490778-153490778	0.001	rs11948898	C	G	0.017	7.33E-03	0.008	2.00E-06	1.06E-08
	5	1	chr5:164585515-164585515	0.001	rs4400143	T	C	-0.017	1.26E-02	-0.009	1.00E-06	2.37E-08
	6	49	chr6:45017228-45328265	311.038	rs12205860	A	G	-0.015	2.63E-02	-0.010	1.90E-07	2.44E-09
	7	1	chr7:100774705-100774705	0.001	rs2227666	A	G	0.053	9.76E-05	-0.020	5.90E-07	7.26E-09
	9	1	chr9:104415321-104415321	0.001	rs1323425	C	T	-0.013	3.42E-02	-0.009	6.00E-07	1.74E-08
	10	1	chr10:10269019-10269019	0.001	rs7907578	T	G	0.042	3.08E-04	-0.015	1.30E-06	4.51E-08
	11	1	chr11:66568943-66568943	0.001	rs682842	T	C	-0.027	4.60E-05	-0.007	1.20E-04	4.54E-08
	13	1	chr13:111603363-111603363	0.001	rs7998604	C	A	-0.021	3.66E-03	-0.009	1.20E-05	3.78E-08
	13	1	chr13:98091008-98091008	0.001	rs1304392	C	T	-0.014	3.79E-02	-0.010	1.60E-07	8.30E-09
	14	1	chr14:60171047-60171047	0.001	rs9323353	G	A	0.027	4.80E-05	-0.009	7.80E-07	1.09E-08
	16	2	chr16:54120330-54123512	3.183	rs10492872	G	T	0.015	2.76E-02	0.009	1.30E-06	1.84E-08
	18	6	chr18:73122598-73147492	24.895	rs11660753	A	G	-0.016	2.04E-02	-0.009	4.90E-07	1.74E-08

**Table 1D:** Novel shared genomic regions shared between choice for instant coffee and T2DM or T2DM-related phenotypes.

T2DM/related phenotypes	CHR	N	POS	KB	Index SNP	A1	A2	Coffee type		T2DM/related phenotype		CPASSOC
								beta	p	beta	p	p
T2DM	1	1	chr1:112277693-112277693	0.001	rs127204	C	A	0.0152	4.32E-03	0.0320	1.00E-06	3.45E-08
	1	6	chr1:26726127-26743903	17.777	rs113466616	A	G	0.0170	2.12E-03	0.0350	1.80E-07	5.92E-09
	1	1	chr1:229594310-229594310	0.001	rs10916480	G	A	0.0161	3.49E-02	0.0510	6.10E-08	1.27E-08
	2	1	chr2:165770457-165770457	0.001	rs16849922	C	A	-0.0122	2.40E-02	0.0330	3.30E-07	3.45E-08
	2	4	chr2:161354368-161368808	14.441	rs79523138	G	A	-0.0187	1.96E-02	0.0540	5.20E-08	3.05E-09
	4	17	chr4:157676228-157745957	69.73	rs4691382	G	A	-0.0150	1.33E-02	-0.0400	9.80E-08	6.78E-09
	5	1	chr5:59398807-59398807	0.001	rs73759013	C	A	-0.0188	4.67E-03	-0.0410	3.10E-07	2.53E-08
	6	3	chr6:153367613-153391618	24.006	rs7767938	C	T	0.0192	1.55E-03	0.0360	9.60E-07	2.77E-08
	7	2	chr7:104415434-104420060	4.627	rs4460308	C	T	0.0176	1.23E-03	0.0330	1.00E-06	1.63E-08
	7	1	chr7:28222877-28222877	0.001	rs139048357	A	C	0.0566	1.49E-02	-0.1600	4.60E-07	1.41E-08
	7	1	chr7:74134911-74134911	0.001	rs35005436	C	T	0.0174	1.52E-02	0.0470	2.50E-07	3.08E-08
	8	4	chr8:19780310-19808131	27.822	rs113023641	A	G	-0.0212	4.70E-03	-0.0480	1.50E-07	1.02E-08
	9	1	chr9:19093017-19093017	0.001	rs10963961	G	C	-0.0116	4.68E-02	0.0360	4.40E-07	4.34E-08
	9	8	chr9:97772285-97795176	22.892	rs10993460	C	T	0.0238	9.79E-03	-0.0580	3.00E-07	5.10E-09
	11	3	chr11:45875392-45877688	2.297	rs7951225	T	A	-0.0155	1.70E-02	-0.0410	2.50E-07	2.89E-08
	12	19	chr12:4237568-4257553	19.986	rs113966250	G	C	-0.0385	3.17E-02	-0.1200	7.00E-08	2.04E-09
	14	3	chr14:69446960-69447386	0.427	rs8016537	A	G	0.0164	1.81E-03	0.0310	1.00E-06	3.22E-08
	14	2	chr14:101309692-101309759	0.068	rs2295388	A	G	0.0154	1.56E-02	-0.0390	3.70E-07	1.75E-08
	17	1	chr17:37402280-37402280	0.001	rs2100651	A	C	-0.0198	9.53E-04	-0.0350	2.00E-06	4.62E-08
	17	2	chr17:17612187-17739163	126.977	rs77745346	T	C	-0.0234	4.17E-02	0.0700	1.60E-07	9.42E-09
	19	9	chr19:13139616-13212025	72.41	rs12151248	T	C	0.0274	7.43E-04	-0.0520	4.10E-07	1.18E-09
BMI	3	1	chr3:66668365-66668365	0.001	rs11128158	G	A	-0.012	3.40E-02	0.010	1.10E-07	7.94E-09
	4	2	chr4:81194090-81199966	5.877	rs3796606	G	A	0.016	3.52E-03	-0.008	2.70E-06	4.73E-08
	7	2	chr7:104435213-104441909	6.697	rs2470961	A	G	0.020	2.02E-04	0.008	4.60E-06	3.28E-08
	10	4	chr10:124929763-124947286	17.524	rs12218544	C	G	0.019	2.05E-02	-0.014	1.40E-07	4.29E-09
	12	1	chr12:117630765-117630765	0.001	rs4492907	C	T	0.011	3.13E-02	0.009	1.00E-07	2.34E-08
	12	6	chr12:84242417-84306965	64.549	rs922160	G	A	0.016	2.76E-03	0.009	3.90E-07	1.93E-08
	16	1	chr16:54120330-54120330	0.001	rs10492872	G	T	-0.013	1.75E-02	0.009	1.30E-06	2.43E-08
	17	1	chr17:35061859-35061859	0.001	rs6607342	C	T	0.017	2.53E-02	0.013	1.20E-07	4.55E-08
	18	1	chr18:73122598-73122598	0.001	rs11660753	A	G	0.013	2.18E-02	-0.009	4.90E-07	4.22E-08
	20	5	chr20:50403227-50505843	102.617	rs6021437	C	T	-0.016	4.41E-03	-0.010	9.90E-08	5.73E-09
	20	1	chr20:8352693-8352693	0.001	rs6086425	G	A	0.016	2.16E-02	-0.011	3.30E-07	1.10E-08

POS, position of shared genomic regions; A1, effect allele of index SNP; A2, non-effect allele of index SNP; CPASSOC, cross-phenotype association analysis using R package “CPASSOC”; T2DM, type 2 diabetes mellitus; BMI, body mass index.

In summary, there were 304 shared genomic regions between total amount of coffee consumed and BMI. Choice for decaffeinated coffee had 75 shared genetic regions with T2DM, 238 shared genetic regions with BMI. Choice for instant coffee and T2DM, BMI, FG, FI, HOMA-IR, had 111, 260, 4, 1, 1 shared regions, respectively. Choice for ground coffee shared the most genomic regions with T2DM and related phenotypes, including 136 shared with T2DM, 88 shared with T2DM (adjusted for BMI), 321 shared with BMI, 15 shared with FG, 7 shared with FI, 10 shared with HOMA-IR.

One of the most important findings of this analysis was the novel genomic regions which had not been reported by previous studies. We identified 134 novel regions totally. For example, we found that rs10492872 located on chromosome 16 was the shared site of choice for instant coffee and choice for ground coffee and BMI, and the effects of this locus on choice for instant coffee and ground coffee were opposite, similar to rs11660753 on chromosome 18. Another SNP, rs4988483, also located on chromosome 16, was the shared site of choice for ground coffee and T2DM (adjusted and unadjusted for BMI), suggesting that this SNP may affect both choice of ground coffee and risk of T2DM through a non-BMI-mediated pathway. Further analysis of these sites will help to understand the association between coffee and T2DM.

The discovery of these shared sites suggested that the genetic correlations between coffee intake or choice for different coffee types and T2DM were likely to be driven by these shared regions. Further research on these regions will help to gain a deeper understanding of the pathogenesis of T2DM and its association with coffee.

### 3.3 KEGG and GO Enrichment Analysis

The KEGG pathways and GO terms enriched by shared genes are shown in **Supplementary Figures 2–8**. We performed enrichment analysis on the four shared gene sets of total coffee intake/choice for different coffee types (decaffeinated coffee, ground coffee, instant coffee) and T2DM or related phenotypes. In the KEGG analysis, in addition to total coffee intake, the remaining three gene sets all showed significant enrichment in allograft rejection (hsa05330), autoimmune thyroid disease (hsa05320), Human T-cell leukemia virus 1 infection (hsa05166), and viral myocarditis (hsa05416).

In GO analysis, all four gene sets were significantly enriched in the following five biological processes related to antigen processing: antigen processing and presentation (GO:0019882), antigen processing and presentation of endogenous peptide antigen (GO:0002483), antigen processing and presentation of endogenous peptide antigen *via* MHC class I (GO:0019885), antigen processing and presentation of exogenous peptide antigen (GO:0002478), antigen processing and presentation of peptide antigen (GO: 0048002).

### 3.4 MR

**Figure 2:**
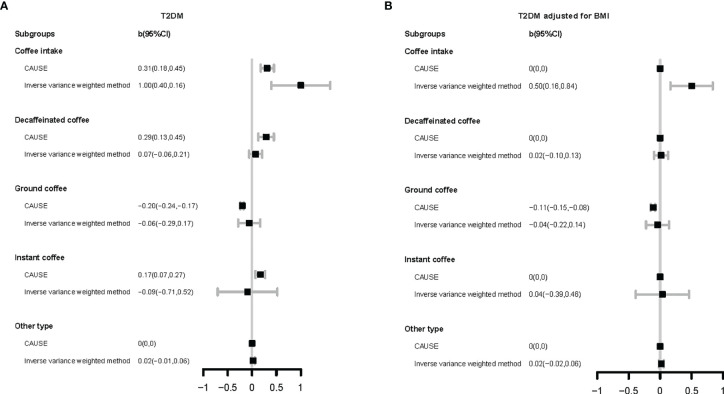
Forest plot for Mendelian randomization results. The vertical axis represents different coffee types and different method. The horizontal axis represents the size of causal effect estimation. T2DM, type 2 diabetes mellitus; BMI, body mass index. CAUSE, causal analysis using summary effect estimates (a mendelian randomization method); CI, confidence interval.

We used eight different methods to estimate the genetically predicated causal effects of coffee intake and coffee type choice on T2DM or related phenotypes. The results are given in **Supplementary Tables 21–25** and shown graphically in **Figure 2**.

For total coffee intake, its harmful associations with T2DM (b _CAUSE_: 0.31, p-value _CAUSE_: 3.30×10^-2^) and BMI (b _CAUSE_: 0.36, p-value _CAUSE_: 1.8×10^-5^) were found by seven methods (except MR-Egger regression). Even after adjusting BMI, its enhancement of T2DM risk was also reflected in the results of six models (except CAUSE and MR-Egger regression).

Analysis of different coffee types indicated various results. Compared with other types of coffee, choice for decaffeinated coffee was found to increase the risk of T2DM (CAUSE; b _CAUSE_: 0.29, p-value _CAUSE_: 2.30×10^-2^) and BMI (IVW, simple median, weighted median, penalized weighted median) by some methods. In the research of instant coffee, the results obtained by different methods were not completely consistent. CAUSE’s result supported the view that choice for instant coffee could increase the risk of T2DM (b _CAUSE_: 0.17, p-value _CAUSE_: 2.90×10^-2^) and this causality was mediated by BMI, but weighted median, penalized weighted median, simple mode, and weighted mode showed the opposite result. Different from the above two types of coffee, choice for ground coffee showed a protective association with glucose homeostasis. It was indicated to decrease BMI (b _CAUSE_: -0.08, p-value _CAUSE_: 6.50×10^-5^) by CAUSE. Regardless of whether BMI was adjusted or not, the results of most methods showed that choice for ground coffee can reduce the risk of T2DM (except IVW and MR-Egger regression; T2DM: b _CAUSE_: -0.2, p-value _CAUSE_: 4.70×10^-10^; T2DM adjusted for BMI: b _CAUSE_: -0.11, p-value _CAUSE_: 4.60×10^-5^).

## 4 Discussion

In the present study, we found that genetically proxied total coffee intake, choice for decaffeinated or instant coffee were significantly associated with increased T2DM risk, whereas genetically proxied choice for ground coffee was associated with decreased risk. We have identified the genes shared by these trait pairs and further determined the biological processes and pathways that these genes were enriched in, and the results indicated the association between coffee intake and T2DM was likely mediated by immune system. This is of great significance for a better understanding of the impact of coffee on T2DM.

In the genetic association analysis, our results showed that those opting for ground coffee (include espresso, filter etc) were less genetically susceptible to T2DM, while those who tended to choose instant coffee or decaffeinated coffee (any type) were more genetically susceptible to these risks (**Figure 1**). Because of the heterogeneity among the consumers of different coffee types showed above, we suggested that combining the data of different coffee beverage consumers in statistical analyses should be done with caution.

We have identified many shared genomic regions between coffee intake or choice for different coffee types and T2DM or related phenotypes (**Supplementary Tables 7–20**). Some of these regions have been discovered in GWAS of single trait, but a considerable part of them has never been discovered till now (**Tables 1A–D**). These regions should be paid more attention because they suggest common unknown pathways which may affect both phenotypes and these regions may partly account for the “missing heritability” (35) in mono-phenotype GWAS.

The results of GO enrichment analysis showed that shared genes were significantly enriched in the biological processes related to antigen processing (**Supplementary Figures 2, 4, 6, 8**). This result suggests that the association between coffee intake and T2DM is likely to be related to immune regulation. The KEGG enrichment results also supported this inference (**Supplementary Figures 3, 5, 7**). Moreover, in the cross-phenotype association study, the notable identified SNP rs10492872 mapped to *FTO* is shared by two trait pairs of ground coffee/BMI and instant coffee/BMI, and its effects on ground coffee and instant coffee are in opposite directions, so as rs11660753 mapped to *SMIM21* (**Tables 1C, D**)*. FTO* gene is the first candidate gene of obesity, and it is also related to a variety of other phenotypes, such as alcohol intake (36), C-reactive protein levels (37), etc. A recent study has found that *FTO* can help cancer cells escape immune surveillance (38). These findings suggest that *FTO* is likely to participate in immune activities. Compared with *FTO*, there is less research on *SMIM21*, but epidemiological studies have also found its significant association with rheumatoid arthritis (39). The above research results suggest that the relationship between coffee intake and T2DM and the differences among various coffee type drinkers are likely to be related to the immune system.

In our MR analysis, we used eight different methods for MR research, with their own characteristics (**Figure 2**, **Supplementary Tables 21–25**). Heterogeneity test showed that heterogeneity was common (**Supplementary Tables 26–30**), which means that the IVs selected according to the conventional principles may be invalid or at least partially invalid. Under this circumstance, the results obtained by IVW may be problematic (40). The test of the intercept term of MR-Egger regression (33) showed that the horizontal pleiotropy did not exist or had reached equilibrium (**Supplementary Table 31**). However, it should be noted that MR-Egger regression can only test the unrelated pleiotropy, that is, the horizontal pleiotropy of IVs on the outcome is not related to confounding factors, but cannot be used to identify related pleiotropy. Our genetic correlation results showed that these phenotypes had extensive genetic correlations (**Figure 1**), which suggested that related pleiotropy was likely to exist. The median/mode method (41) has lower requirements for IVs, but considering that it uses little information, the results need to be carefully understood. As a newly proposed method, the CAUSE method (34) can deal with related and unrelated pleiotropy to identify causal models and shared models, but the validity of its results needs to be tested in more applications. In short, we recommend that MR results should be obtained from a combination of different methods such as we did here to address relationships between lifestyle factors and health optimally.

We have noticed that there have been MR studies on coffee intake and the risk of T2DM published (12, 14, 42). Among them, the results of the MR study by Shuai Yuan et al. (12) was similar to our results, they found that coffee intake could increase the risk of T2DM, but this effect disappeared after adjusting for BMI. Whereas the remaining two studies (14, 42) found no significant effect of coffee intake on the risk of T2DM. Compared with these researches, our study has several outstanding advantages. First, in addition to total coffee intake, we also analyzed the association between the choice for different coffee types and T2DM, and found significant differences between various coffee type drinkers. This is not considered by the previous MR studies on coffee intake and T2DM. Second, instead of using only a few variants related to caffeine metabolism, we used more variants as IVs to achieve a greater proportion of explanation for the variance of coffee intake. Finally, we used multiple MR methods with different advantages to overcome the limitations of a single method. Thus, we believe that our research is more reliable and meaningful.

Both the results of genetic correlation study and MR analysis showed that choice for ground coffee was associated with lower risk of T2DM, compared to instant coffee and decaffeinated coffee. This indicates that the content of coffee’s beneficial cardiovascular components is different in various coffee types. This is consistent with the results of the laboratory investigation. A study investigating the content of CGAs and caffeine in 83 commercially available coffee species found that unblended ground coffee had the highest CGAs content and lowest mean caffeine/CGAs ratio (16). Study on total phenol content has reached similar conclusions (4). In instant coffee, the lower beneficial effects of chlorogenic acid and total phenols are likely to be offset by the harmful effects of added sugar, creamer, and other ingredients, as the results of observational studies on instant coffee showed (43).

When we directly analyzed total coffee intake without distinguishing between types, we found significant positive associations. This may be because in the UKB participants, compared with ground coffee, people who drink instant coffee or decaffeinated coffee account for a larger proportion. Actual data support this inference (decaffeinated coffee: N=64,717, instant coffee: N=185,482; ground coffee: N=73,906; other type of coffee: N=5,566). Although we are very clear about the cardiovascular beneficial effects of certain components in coffee, previous studies on the association between coffee intake and cardiovascular risks have not reached a consistent result. Some studies thought that moderate coffee drinking can benefit cardiovascular health (44), but there are also research suggesting the harmful effects of coffee (12, 13). Our results suggest a possible reason for this phenomenon. We suggest that research on the health effects of coffee should distinguish between coffee types.

There are several notable strengths of our work. First, this is the first article to simultaneously study the genetic and potential causal relationship between coffee intake and T2DM. We identified many shared genomic regions and found significant causal relationship, which provided new insights into the mechanism of their associations. In addition to the amount of total coffee intake, we also studied the relationship between choice for different types of coffee and T2DM. The differences in the results suggest the importance of distinguishing different types of coffee in coffee research. In addition to T2DM, we have also investigated other T2DM-related phenotypes (BMI, FG, FI, HOMA-IR, HOMA-β), which will facilitate a more thorough understanding of the association between coffee and T2DM. The GWAS summary statistics selected for our study are all from large-scale and high-quality GWAS. We identify many shared loci and found many significant causal relationships that have not been found in previous less-powerful MR studies.

Our research still has some areas that can be further improved. First, for different types of coffee, it should be noted that we are studying the relationship between choosing this type of coffee and T2DM, but not the amount of this type of coffee consumed. In the future, if relevant data is available, the association between the consumption amount of each type of coffee and T2DM can be further studied. Second, although it is observed that there is a great difference between the results of instant coffee and ground coffee, our research results still cannot answer whether this difference is due to the composition difference caused by the processing or the usual addition of saccharin and other additives in instant coffee. Later, we will further analyze whether the preference of adding milk/cream/sugar to coffee will affect its association with T2DM. We believe that these sites will be much useful for studying the pathogenesis of T2DM and its relationship with coffee.

In summary, we have shown that choice for ground coffee (include espresso, filter etc) has extensive significant negative genetic correlation with T2DM and related phenotypes. MR analysis using all variants indicated genetically proxied choice for ground coffee can decrease BMI and the risk of T2DM, while other types of coffee may increase the risk of T2DM. This study provides new insights and evidence for the health effect of coffee. The results of different coffee types suggests that research on coffee’s health effect should pay more attention to distinguishing between coffee types.

## Data Availability Statement

The original contributions presented in the study are included in the article/**Supplementary Material**. Further inquiries can be directed to the corresponding authors.

## Author Contributions

XW, JJ, and TH designed the study. XW performed the statistical analysis. XW wrote the manuscript. All authors helped interpret the data, reviewed, and edited the final paper, and approved the submission.

## Funding

The study was supported by grants from the Peking University Start-up Grant (71013Y2114), High-performance Computing Platform of Peking University and Beijing Technology and Business University Grant(88442Y0033). The funding organization had no role in the preparation of the manuscript.

## Conflict of Interest

The authors declare that the research was conducted in the absence of any commercial or financial relationships that could be construed as a potential conflict of interest.

## Publisher’s Note

All claims expressed in this article are solely those of the authors and do not necessarily represent those of their affiliated organizations, or those of the publisher, the editors and the reviewers. Any product that may be evaluated in this article, or claim that may be made by its manufacturer, is not guaranteed or endorsed by the publisher.
