# Supplementary material for: Coffee Types and Type 2 Diabetes Mellitus: Large-Scale Cross-Phenotype Association Study and Mendelian Randomization Analysis
Source: Front Endocrinol (Lausanne). 2022 Feb 11;13:818831. doi: 10.3389/fendo.2022.818831 (PMC8873575; doi:10.3389/fendo.2022.818831)
Supplement: Supplementary file 1 [file DataSheet_1.docx]

Supplementary Figure 1. Flowchart

The flowchart of the present study. T2DM: type 2 diabetes mellitus; BMI: body mass index; FG: fasting glucose; FI: fasting insulin; HOMA-IR: insulin resistance; HOMA-β: beta-cell function; GWAS: genome-wide association study; LD: Linkage disequilibrium; KEGG: Kyoto Encyclopedia of Genes and Genomes; GO: Gene Ontology.

Supplementary Figure 2. Enriched GO terms of shared genes between coffee intake and T2DM (including T2DM-related phenotypes) (top 10).


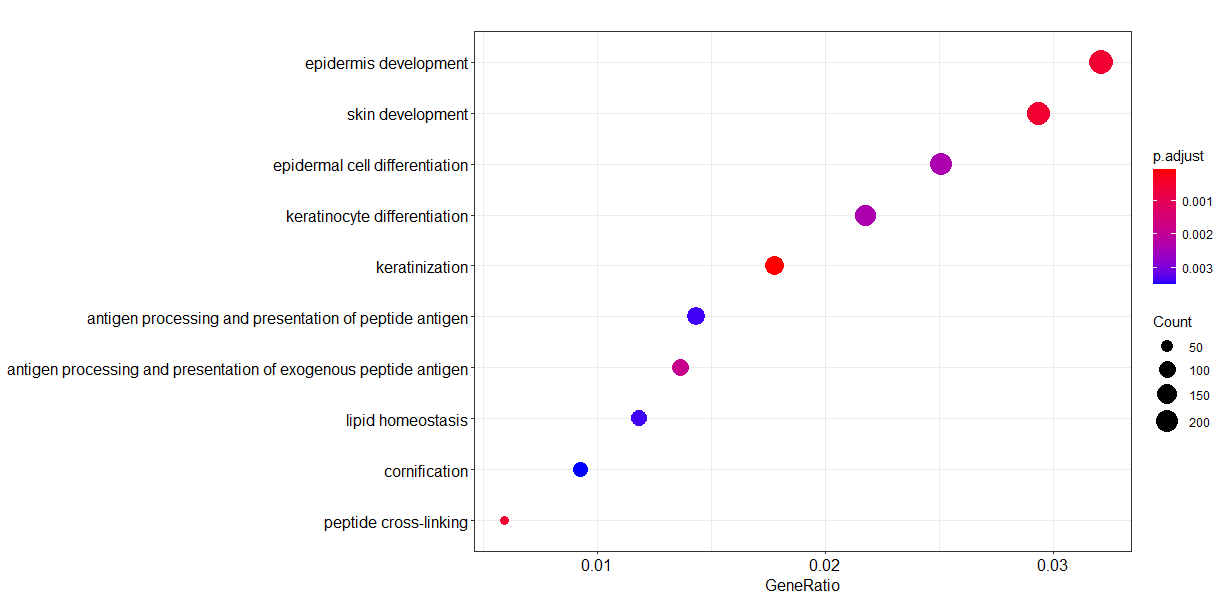


GO: Gene Ontology; T2DM: type 2 diabetes mellitus.

Supplementary Figure 3. Enriched KEGG pathways of shared genes between choice of decaffeinated coffee and T2DM (including T2DM-related phenotypes) (top 10).


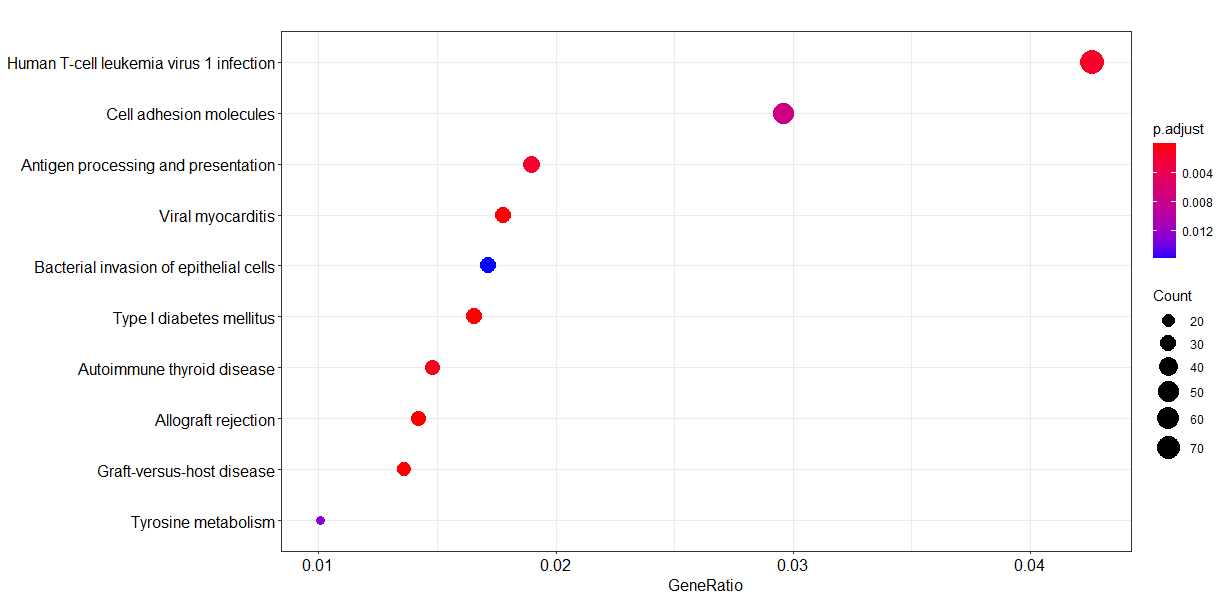


KEGG: Kyoto Encyclopedia of Genes and Genomes; T2DM: type 2 diabetes mellitus.

Supplementary Figure 4. Enriched GO terms of shared genes between choice of decaffeinated coffee and T2DM (including T2DM-related phenotypes) (top 10).


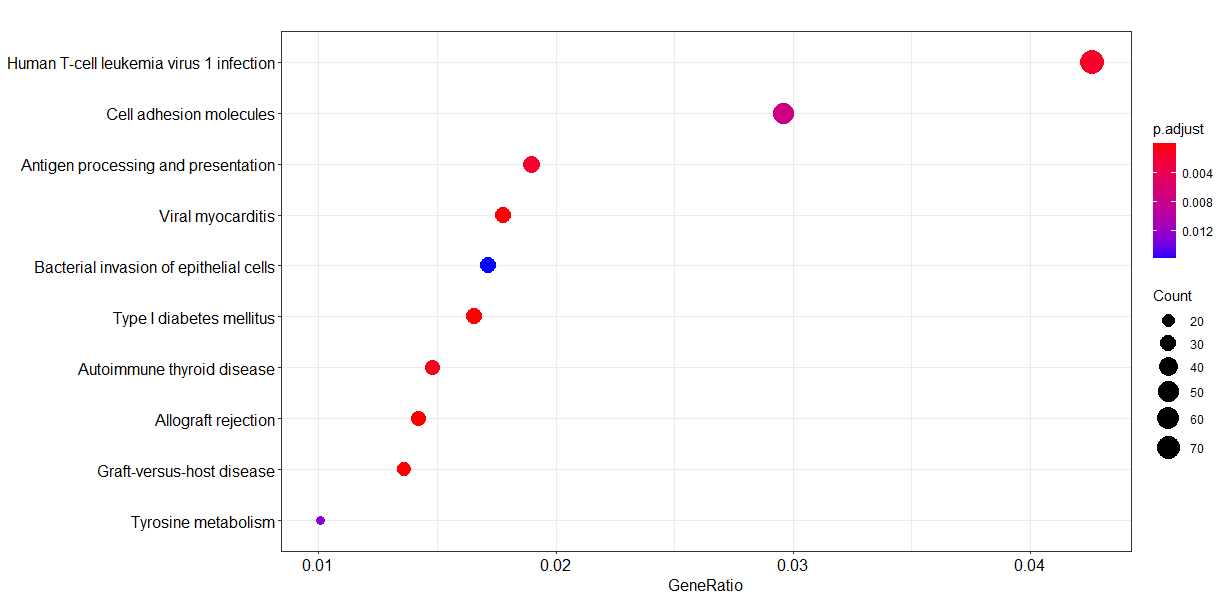


GO: Gene Ontology; T2DM: type 2 diabetes mellitus.

Supplementary Figure 5. Enriched KEGG pathways of shared genes between choice of ground coffee and T2DM (including T2DM-related phenotypes) (top 10).


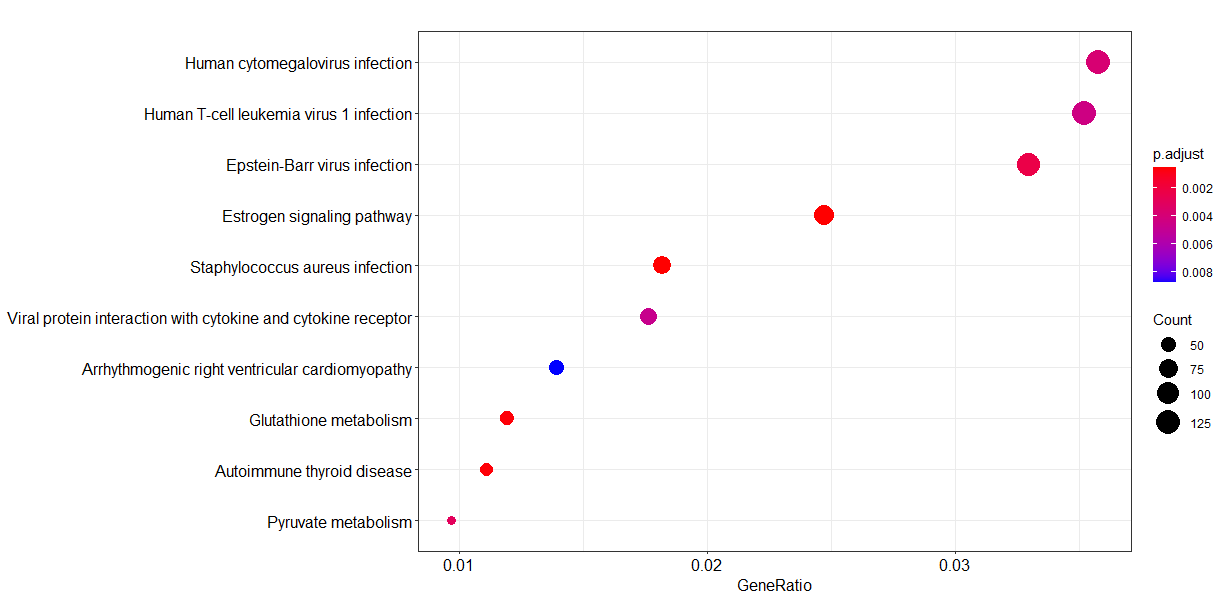


KEGG: Kyoto Encyclopedia of Genes and Genomes; T2DM: type 2 diabetes mellitus.

Supplementary Figure 6. Enriched GO terms of shared genes between choice of ground coffee and T2DM (including T2DM-related phenotypes) (top 10).


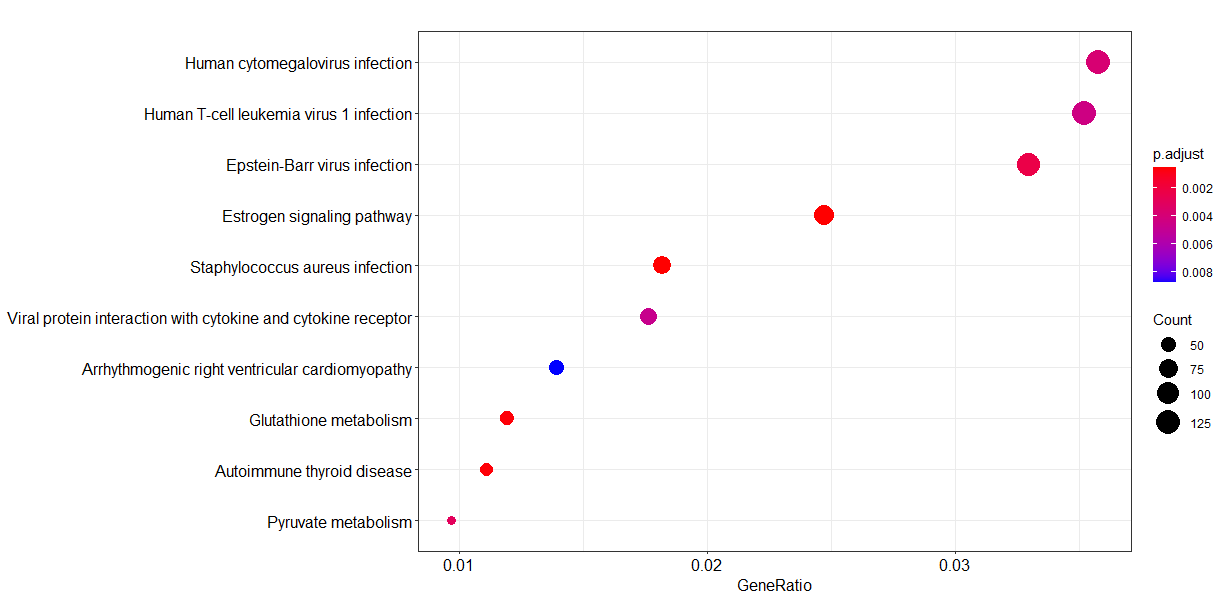


GO: Gene Ontology; T2DM: type 2 diabetes mellitus.

Supplementary Figure 7. Enriched KEGG pathways of shared genes between choice of instant coffee and T2DM (including T2DM-related phenotypes) (top 10).


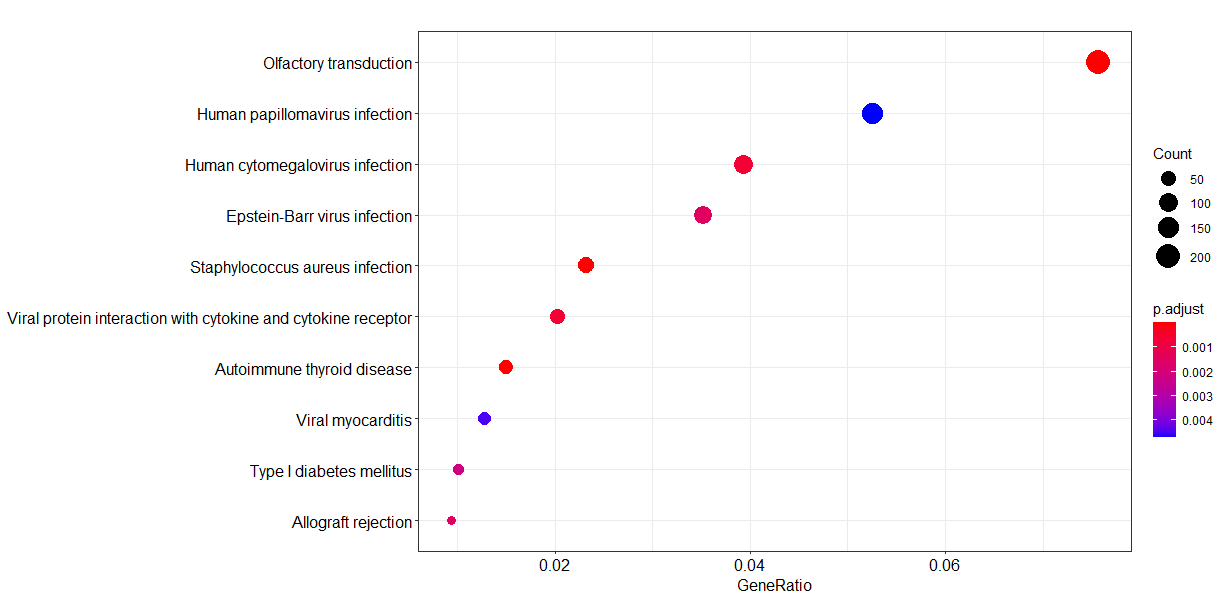


KEGG: Kyoto Encyclopedia of Genes and Genomes; T2DM: type 2 diabetes mellitus.

Supplementary Figure 8. Enriched GO terms of shared genes between choice of instant coffee and T2DM (including T2DM-related phenotypes) (top 10).


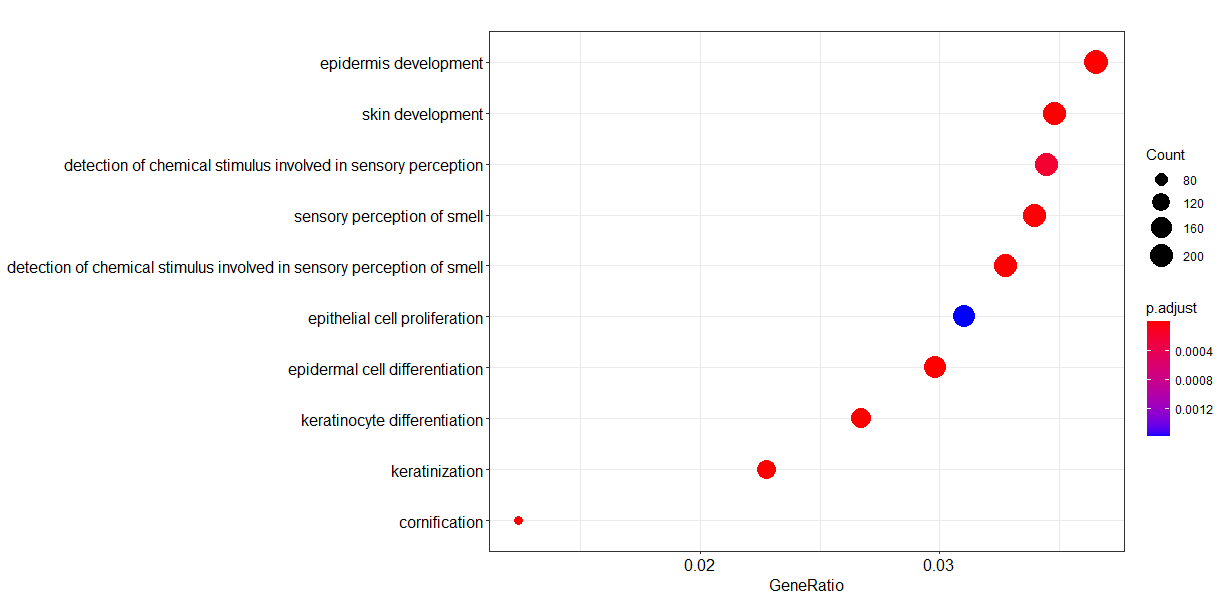


GO: Gene Ontology; T2DM: type 2 diabetes mellitus.

Supplementary Table 1. Data Source

| Phenotype | N-total | Publication | Link |
| --- | --- | --- | --- |
| Coffee intake  Choice for different coffee types | 389,665/ 329,671 | Nealelab | http://www.nealelab.is/uk-biobank/ |
| T2DM/T2DM(bmiadj) | 898,130 | Fine-mapping type 2 diabetes loci to single-variant resolution using high-density imputation and islet-specific epigenome maps | https://pubmed.ncbi.nlm.nih.gov/30297969/ |
| BMI | ∼700,000 | Meta-analysis of genome-wide association studies for height and body mass index in ∼700000 individuals of European ancestry | https://pubmed.ncbi.nlm.nih.gov/30124842/ |
| FG/FI/HOMA-IR/HOMA-β | 46,186/38,238//37,037/36,466 | New genetic loci implicated in fasting glucose homeostasis and their impact on type 2 diabetes risk | https://pubmed.ncbi.nlm.nih.gov/20081858/ |

T2DM: type 2 diabetes mellitus; BMI: body mass index; FG: fasting glucose; FI: fasting insulin; HOMA-IR: insulin resistance; HOMA-β: beta-cell function.

Supplementary Table 2. Genetic correlation of coffee intake and T2DM as well as T2DM-related phenotypes.

| T2DM and related phenotypes | Genetic correlation | P-value |
| --- | --- | --- |
| T2DM | 0.02 | 4.78E-01 |
| T2DM (adjusted for BMI) | -0.07 | 1.52E-02 |
| BMI | 0.26 | 1.12E-30 |
| FG | -0.01 | 8.70E-01 |
| FI | 0.05 | 4.22E-01 |
| HOMA-IR | 0.09 | 1.65E-01 |
| HOMA-β | 0.14 | 2.43E-02 |

T2DM: type 2 diabetes mellitus; BMI: body mass index; FG: fasting glucose; FI: fasting insulin; HOMA-IR: insulin resistance; HOMA-β: beta-cell function.

Supplementary Table 3. Genetic correlation of choice for Decaffeinated coffee and T2DM as well as T2DM-related phenotypes.

| T2DM and related phenotypes | Genetic correlation | P-value |
| --- | --- | --- |
| T2DM | 0.15 | 1.00E-04 |
| T2DM (adjusted for BMI) | 0.08 | 5.73E-02 |
| BMI | 0.15 | 2.96E-06 |
| FG | -0.04 | 5.94E-01 |
| FI | 0.07 | 4.90E-01 |
| HOMA-IR | 0.05 | 6.33E-01 |
| HOMA-β | 0.10 | 2.38E-01 |

T2DM: type 2 diabetes mellitus; BMI: body mass index; FG: fasting glucose; FI: fasting insulin; HOMA-IR: insulin resistance; HOMA-β: beta-cell function.

Supplementary Table 4. Genetic correlation of choice for Instant coffee and T2DM as well as T2DM-related phenotypes.

| T2DM and related phenotypes | Genetic correlation | P-value |
| --- | --- | --- |
| T2DM | 0.11 | 1.00E-03 |
| T2DM (adjusted for BMI) | 0.05 | 1.48E-01 |
| BMI | 0.15 | 1.73E-07 |
| FG | 0.21 | 1.00E-03 |
| FI | 0.36 | 6.40E-06 |
| HOMA-IR | 0.40 | 4.86E-06 |
| HOMA-β | 0.16 | 3.15E-02 |

T2DM: type 2 diabetes mellitus; BMI: body mass index; FG: fasting glucose; FI: fasting insulin; HOMA-IR: insulin resistance; HOMA-β: beta-cell function.

Supplementary Table 5. Genetic correlation of choice for Ground coffee and T2DM as well as T2DM-related phenotypes.

| T2DM and related phenotypes | Genetic correlation | P-value |
| --- | --- | --- |
| T2DM | -0.18 | 2.81E-13 |
| T2DM (adjusted for BMI) | -0.10 | 3.00E-04 |
| BMI | -0.21 | 6.40E-26 |
| FG | -0.19 | 9.39E-05 |
| FI | -0.36 | 6.88E-08 |
| HOMA-IR | -0.38 | 2.72E-08 |
| HOMA-β | -0.18 | 1.80E-03 |

T2DM: type 2 diabetes mellitus; BMI: body mass index; FG: fasting glucose; FI: fasting insulin; HOMA-IR: insulin resistance; HOMA-β: beta-cell function.

Supplementary Table 6. Genetic correlation of choice for other types of coffee and T2DM as well as T2DM-related phenotypes.

| T2DM and related phenotypes | Genetic correlation | P-value |
| --- | --- | --- |
| T2DM | 0.16 | 6.50E-03 |
| T2DM (adjusted for BMI) | 0.14 | 3.28E-02 |
| BMI | 0.07 | 1.40E-01 |
| FG | 0.24 | 3.15E-02 |
| FI | 0.07 | 6.24E-01 |
| HOMA-IR | 0.07 | 6.58E-01 |
| HOMA-β | -0.05 | 7.22E-01 |

T2DM: type 2 diabetes mellitus; BMI: body mass index; FG: fasting glucose; FI: fasting insulin; HOMA-IR: insulin resistance; HOMA-β: beta-cell function.

Supplementary Table 7. Shared genetic loci of coffee intake and body mass index (BMI) identified by cross-phenotype association analysis (top 20).

| Index SNP | Chr | N | Pos | Kb | A1 | A2 | coffee intake | | BMI | | P |
| --- | --- | --- | --- | --- | --- | --- | --- | --- | --- | --- | --- |
|  |  |  |  |  |  |  | Beta | P | Beta | P |  |
| rs8047395 | 16 | 82 | chr16:53550898..53908657 | 357.76 | A | G | 0.0135 | 9.96E-16 | 0.0642 | 0.00E+00 | 0.00E+00 |
| rs6567160 | 18 | 350 | chr18:52473167..63458462 | 10985.3 | C | T | 0.0168 | 2.05E-17 | 0.0550 | 1.80E-178 | 2.12E-196 |
| rs13021737 | 2 | 266 | chr2:249092..6236608 | 5987.52 | G | A | 0.0155 | 3.68E-12 | 0.0574 | 7.50E-157 | 3.48E-175 |
| rs2472297 | 15 | 225 | chr15:66365220..77904415 | 11539.2 | T | C | 0.0461 | 3.11E-131 | 0.0122 | 1.40E-10 | 1.80E-139 |
| rs543874 | 1 | 172 | chr1:173457834..181056160 | 7598.33 | G | A | 0.0116 | 1.96E-08 | 0.0475 | 1.20E-122 | 1.97E-132 |
| rs4410790 | 7 | 141 | chr7:17109704..27248891 | 10139.2 | C | T | 0.0392 | 8.19E-112 | 0.0099 | 4.10E-08 | 1.39E-118 |
| rs10182181 | 2 | 122 | chr2:23801620..32954020 | 9152.4 | G | A | 0.0039 | 1.92E-02 | 0.0325 | 6.70E-90 | 4.67E-97 |
| rs12641981 | 4 | 79 | chr4:36813105..45224500 | 8411.4 | T | C | 0.0082 | 1.16E-06 | 0.0324 | 8.00E-86 | 1.82E-96 |
| rs943005 | 6 | 315 | chr6:41738158..53958521 | 12220.4 | T | C | 0.0098 | 1.22E-05 | 0.0415 | 2.70E-83 | 6.64E-92 |
| rs11030104 | 11 | 70 | chr11:27541623..32160524 | 4618.9 | G | A | -0.0074 | 4.01E-04 | -0.0393 | 2.00E-83 | 7.11E-91 |
| rs2307111 | 5 | 201 | chr5:74242852..75222161 | 979.31 | C | T | -0.0040 | 1.86E-02 | -0.0265 | 1.60E-58 | 9.20E-65 |
| rs1993709 | 1 | 22 | chr1:66362971..80824657 | 14461.7 | G | A | 0.0044 | 3.62E-02 | 0.0331 | 1.90E-57 | 1.02E-58 |
| rs8025790 | 15 | 45 | chr15:60878030..68201422 | 7323.39 | T | G | -0.0045 | 2.37E-02 | -0.0291 | 1.10E-50 | 1.76E-55 |
| rs3810291 | 19 | 34 | chr19:45389596..51812034 | 6422.44 | A | G | 0.0084 | 3.12E-06 | 0.0274 | 2.10E-52 | 8.18E-55 |
| rs12446632 | 16 | 158 | chr16:19708196..29910725 | 10202.5 | A | G | -0.0110 | 4.56E-06 | -0.0352 | 2.90E-50 | 6.17E-51 |
| rs2814992 | 6 | 186 | chr6:31082361..42672416 | 11590.1 | G | A | 0.0060 | 7.27E-04 | 0.0245 | 4.10E-45 | 3.32E-49 |
| rs3814424 | 5 | 139 | chr5:80842034..96138861 | 15296.8 | T | C | 0.0173 | 6.79E-14 | 0.0300 | 8.60E-35 | 2.57E-45 |
| rs17066856 | 18 | 10 | chr18:58033935..63374392 | 5340.46 | C | T | -0.0074 | 1.14E-02 | -0.0385 | 1.50E-42 | 7.47E-45 |
| rs3814883 | 16 | 50 | chr16:20410263..31011183 | 10600.9 | T | C | -0.0037 | 2.97E-02 | 0.0232 | 1.10E-40 | 3.40E-44 |
| rs2820311 | 1 | 36 | chr1:201800868..210306844 | 8505.98 | G | A | -0.0041 | 2.20E-02 | 0.0235 | 4.10E-38 | 1.66E-40 |

Chr: chromosome; Pos: chromosome position; Kb: the length of the shared genomic region; N: number of identified significant SNPs in this region; A1: effect allele; A2: another allele; Beta: effect size in single-phenotype genome-wide association study; P: p value.

Supplementary Table 8. Shared genetic loci of choice for decaffeinated coffee and type 2 diabetes mellitus (T2DM) identified by cross-phenotype association analysis (top 20).

| Index SNP | Chr | N | Pos | Kb | A1 | A2 | decaffeinated coffee | | T2DM | | P |
| --- | --- | --- | --- | --- | --- | --- | --- | --- | --- | --- | --- |
|  |  |  |  |  |  |  | Beta | P | Beta | P |  |
| rs7080591 | 10 | 26 | chr10:114561232..114770860 | 209.629 | T | C | -0.0145 | 2.56E-02 | 0.1200 | 1.10E-73 | 1.24E-81 |
| rs13388242 | 2 | 4 | chr2:227020653..227117850 | 97.198 | A | G | -0.0149 | 2.66E-02 | -0.0810 | 6.30E-33 | 2.01E-35 |
| rs11709077 | 3 | 31 | chr3:12106191..12396955 | 290.765 | A | G | 0.0194 | 4.82E-02 | -0.1100 | 1.60E-27 | 8.00E-31 |
| rs703971 | 10 | 19 | chr10:71462805..80953436 | 9490.63 | C | G | 0.0152 | 2.01E-02 | -0.0700 | 1.10E-25 | 1.93E-28 |
| rs3768321 | 1 | 217 | chr1:39551488..40075894 | 524.407 | T | G | 0.0169 | 3.42E-02 | 0.0850 | 1.30E-26 | 1.01E-27 |
| rs2299620 | 11 | 6 | chr11:2181338..2858295 | 676.958 | T | C | -0.0369 | 3.47E-02 | -0.1900 | 2.50E-26 | 2.20E-27 |
| rs780094 | 2 | 2 | chr2:27541053..27741237 | 200.185 | C | T | -0.0129 | 4.87E-02 | 0.0650 | 1.60E-23 | 5.62E-25 |
| rs2506125 | 9 | 41 | chr9:81364278..84384450 | 3020.17 | A | G | 0.0153 | 1.74E-02 | -0.0600 | 6.20E-20 | 2.72E-22 |
| rs1063192 | 9 | 92 | chr9:21974218..22121349 | 147.132 | A | G | 0.0180 | 4.91E-03 | 0.0580 | 2.90E-19 | 8.01E-22 |
| rs9379084 | 6 | 1 | chr6:7231843..7231843 | 0.001 | A | G | 0.0219 | 3.30E-02 | -0.0970 | 2.30E-20 | 3.41E-20 |
| rs9687846 | 5 | 28 | chr5:53394938..55864102 | 2469.16 | A | G | 0.0175 | 2.70E-02 | 0.0690 | 8.40E-18 | 9.00E-19 |
| rs1377807 | 17 | 60 | chr17:3988150..4229258 | 241.109 | C | G | 0.0135 | 4.92E-02 | 0.0570 | 5.70E-17 | 6.37E-18 |
| rs13107325 | 4 | 95 | chr4:102707791..103956956 | 1249.17 | T | C | 0.1011 | 9.42E-17 | 0.0480 | 3.30E-04 | 3.89E-17 |
| rs2028150 | 2 | 40 | chr2:60589262..65707760 | 5118.5 | G | C | -0.0127 | 4.94E-02 | -0.0520 | 3.10E-15 | 1.68E-16 |
| rs9388490 | 6 | 109 | chr6:126609124..127796326 | 1187.2 | T | C | -0.0160 | 1.27E-02 | 0.0490 | 4.50E-14 | 2.81E-16 |
| rs9820223 | 3 | 34 | chr3:186629289..187755222 | 1125.93 | C | T | -0.0175 | 7.31E-03 | 0.0500 | 8.20E-14 | 3.01E-16 |
| rs11555762 | 11 | 99 | chr11:43634973..43878485 | 243.513 | T | C | 0.0193 | 4.93E-03 | 0.0490 | 1.40E-12 | 9.53E-15 |
| rs340881 | 1 | 3 | chr1:214149028..214223408 | 74.381 | A | T | 0.0156 | 3.26E-02 | 0.0550 | 1.20E-13 | 1.04E-14 |
| rs355828 | 2 | 49 | chr2:165610498..165822558 | 212.061 | T | C | -0.0133 | 4.18E-02 | -0.0480 | 3.40E-13 | 1.98E-14 |
| rs7747095 | 6 | 11 | chr6:43826353..50889848 | 7063.5 | A | C | -0.0172 | 2.30E-02 | 0.0540 | 1.00E-12 | 2.19E-14 |

Chr: chromosome; Pos: chromosome position; Kb: the length of the shared genomic region; N: number of identified significant SNPs in this region; A1: effect allele; A2: another allele; Beta: effect size in single-phenotype genome-wide association study; P: p value.

Supplementary Table 9. Shared genetic loci of choice for decaffeinated coffee and body mass index (BMI) identified by cross-phenotype association analysis (top 20).

| Index SNP | Chr | N | Pos | Kb | A1 | A2 | decaffeinated coffee | | BMI | | P |
| --- | --- | --- | --- | --- | --- | --- | --- | --- | --- | --- | --- |
|  |  |  |  |  |  |  | Beta | P | Beta | P |  |
| rs943005 | 6 | 33 | chr6:43352898..51809617 | 8456.72 | T | C | 0.0181 | 3.25E-02 | 0.0415 | 2.70E-83 | 1.68E-92 |
| rs6265 | 11 | 13 | chr11:27628269..27694241 | 65.973 | T | C | 0.0211 | 9.28E-03 | -0.0412 | 1.00E-86 | 3.53E-91 |
| rs9956279 | 18 | 30 | chr18:53335512..60783211 | 7447.7 | T | C | -0.0138 | 4.69E-02 | 0.0349 | 3.50E-84 | 4.55E-89 |
| rs8049439 | 16 | 70 | chr16:24743657..31370090 | 6626.43 | C | T | 0.0166 | 1.04E-02 | 0.0265 | 1.50E-58 | 3.49E-65 |
| rs13107325 | 4 | 62 | chr4:94413681..103374760 | 8961.08 | T | C | 0.1011 | 9.42E-17 | 0.0470 | 1.10E-47 | 4.64E-65 |
| rs3101336 | 1 | 66 | chr1:72423184..72992580 | 569.397 | C | T | 0.0150 | 2.11E-02 | 0.0264 | 4.60E-57 | 2.16E-57 |
| rs3810291 | 19 | 20 | chr19:47569003..47707427 | 138.425 | A | G | 0.0165 | 1.55E-02 | 0.0274 | 2.10E-52 | 3.59E-55 |
| rs563590 | 2 | 37 | chr2:25301755..27839832 | 2538.08 | G | A | -0.0141 | 2.89E-02 | -0.0245 | 1.30E-45 | 1.59E-49 |
| rs11713193 | 3 | 107 | chr3:43962759..50644134 | 6681.38 | A | G | 0.0149 | 1.89E-02 | 0.0239 | 2.40E-44 | 3.65E-47 |
| rs17201502 | 12 | 13 | chr12:50183874..50285562 | 101.689 | T | C | -0.0132 | 4.88E-02 | 0.0250 | 3.10E-42 | 4.77E-46 |
| rs561634 | 1 | 23 | chr1:173457834..177901741 | 4443.91 | T | A | 0.0134 | 3.74E-02 | 0.0211 | 4.10E-38 | 1.39E-41 |
| rs12429545 | 13 | 137 | chr13:53742128..60730503 | 6988.38 | A | G | 0.0217 | 2.34E-02 | 0.0316 | 9.60E-38 | 2.92E-38 |
| rs7355953 | 3 | 387 | chr3:77596134..94051397 | 16455.3 | C | T | -0.0294 | 1.53E-04 | 0.0224 | 7.40E-29 | 5.66E-34 |
| rs7928523 | 11 | 72 | chr11:43599791..43876435 | 276.645 | T | C | 0.0169 | 1.36E-02 | 0.0213 | 8.10E-31 | 1.28E-33 |
| rs1108682 | 3 | 64 | chr3:88726844..94249591 | 5522.75 | A | T | 0.0145 | 2.22E-02 | -0.0195 | 6.40E-30 | 1.24E-31 |
| rs11880870 | 19 | 4 | chr19:18234588..18830704 | 596.117 | G | A | -0.0131 | 3.95E-02 | -0.0189 | 1.00E-28 | 9.25E-30 |
| rs16952522 | 16 | 27 | chr16:49010797..53861024 | 4850.23 | G | C | 0.0407 | 1.54E-02 | 0.0518 | 1.00E-28 | 2.94E-29 |
| rs10840099 | 11 | 49 | chr11:8568207..13361524 | 4793.32 | C | T | 0.0151 | 2.39E-02 | 0.0182 | 9.90E-27 | 1.20E-27 |
| rs10962110 | 9 | 106 | chr9:9043015..16048164 | 7005.15 | A | G | 0.0134 | 3.68E-02 | -0.0181 | 1.10E-25 | 2.36E-27 |
| rs16851483 | 3 | 13 | chr3:131786554..141335121 | 9548.57 | T | G | 0.0280 | 2.93E-02 | 0.0369 | 3.20E-26 | 7.80E-27 |

Chr: chromosome; Pos: chromosome position; Kb: the length of the shared genomic region; N: number of identified significant SNPs in this region; A1: effect allele; A2: another allele; Beta: effect size in single-phenotype genome-wide association study; P: p value.

Supplementary Table 10. Shared genetic loci of choice for ground coffee and type 2 diabetes mellitus (T2DM) identified by cross-phenotype association analysis (top 20).

| Index SNP | Chr | N | Pos | Kb | A1 | A2 | ground coffee | | T2DM | | P |
| --- | --- | --- | --- | --- | --- | --- | --- | --- | --- | --- | --- |
|  |  |  |  |  |  |  | Beta | P | Beta | P |  |
| rs7899529 | 10 | 71 | chr10:114654837..114916586 | 261.75 | A | G | 0.0152 | 1.75E-02 | 0.1700 | 2.80E-149 | 1.10E-174 |
| rs1421085 | 16 | 170 | chr16:53429173..53848561 | 419.389 | C | T | 0.0279 | 1.70E-05 | 0.1200 | 2.40E-78 | 4.40E-90 |
| rs4930011 | 11 | 12 | chr11:2663891..8694830 | 6030.94 | G | C | 0.0137 | 3.69E-02 | 0.0930 | 6.60E-43 | 8.24E-47 |
| rs4234731 | 4 | 130 | chr4:3243804..6319089 | 3075.29 | G | A | -0.0147 | 2.36E-02 | 0.0870 | 4.40E-40 | 1.65E-43 |
| rs11602873 | 11 | 14 | chr11:65560785..72477815 | 6917.03 | T | A | -0.0171 | 4.92E-02 | -0.1100 | 2.60E-33 | 2.67E-38 |
| rs6483206 | 11 | 5 | chr11:92678397..92701596 | 23.2 | T | C | -0.0132 | 4.94E-02 | 0.0790 | 1.50E-31 | 6.36E-34 |
| rs1215470 | 13 | 5 | chr13:80617309..80706455 | 89.147 | C | T | -0.0142 | 4.39E-02 | -0.0800 | 4.30E-30 | 1.35E-32 |
| rs115246607 | 5 | 16 | chr5:101123995..106377791 | 5253.8 | C | T | 0.0327 | 2.88E-02 | 0.1700 | 5.20E-29 | 1.81E-32 |
| rs849335 | 7 | 3 | chr7:28223990..28256240 | 32.251 | C | T | 0.0139 | 3.80E-02 | -0.0760 | 2.20E-30 | 2.19E-32 |
| rs11709077 | 3 | 87 | chr3:12027240..12473045 | 445.806 | A | G | -0.0224 | 2.25E-02 | -0.1100 | 1.60E-27 | 4.16E-32 |
| rs703976 | 10 | 127 | chr10:71464178..81018948 | 9554.77 | G | C | 0.0183 | 4.03E-03 | -0.0710 | 5.00E-28 | 4.76E-31 |
| rs1870565 | 12 | 164 | chr12:66162603..66379504 | 216.902 | T | C | -0.0480 | 8.29E-06 | 0.1100 | 8.90E-23 | 3.74E-28 |
| rs3768321 | 1 | 99 | chr1:39551488..40073011 | 521.524 | T | G | -0.0199 | 1.30E-02 | 0.0850 | 1.30E-26 | 4.01E-28 |
| rs340882 | 1 | 78 | chr1:205030862..219731273 | 14700.4 | G | C | -0.0134 | 4.15E-02 | 0.0670 | 1.10E-24 | 4.31E-26 |
| rs11759026 | 6 | 237 | chr6:126052359..127167072 | 1114.71 | G | A | 0.0240 | 1.66E-03 | 0.0670 | 1.30E-18 | 6.53E-23 |
| rs9350294 | 6 | 20 | chr6:20639604..26336696 | 5697.09 | C | T | 0.0153 | 2.14E-02 | 0.0620 | 2.50E-20 | 8.62E-23 |
| rs601945 | 6 | 216 | chr6:26295926..32636434 | 6340.51 | G | A | -0.0183 | 2.39E-02 | 0.0800 | 2.70E-21 | 2.67E-22 |
| rs9379084 | 6 | 22 | chr6:6997161..7267313 | 270.153 | A | G | -0.0218 | 3.38E-02 | -0.0970 | 2.30E-20 | 1.21E-20 |
| rs1912980 | 2 | 55 | chr2:60561692..65713102 | 5151.41 | T | C | -0.0144 | 2.46E-02 | -0.0560 | 4.60E-18 | 1.41E-20 |
| rs9668702 | 12 | 141 | chr12:26253557..33435427 | 7181.87 | G | T | -0.0166 | 2.81E-02 | -0.0660 | 8.80E-18 | 3.22E-20 |

Chr: chromosome; Pos: chromosome position; Kb: the length of the shared genomic region; N: number of identified significant SNPs in this region; A1: effect allele; A2: another allele; Beta: effect size in single-phenotype genome-wide association study; P: p value.

Supplementary Table 11. Shared genetic loci of choice for ground coffee and type 2 diabetes mellitus (T2DM) (adjusted for BMI) identified by cross-phenotype association analysis (top 20).

| Index SNP | Chr | N | Pos | Kb | A1 | A2 | ground coffee | | T2DM adjusted for BMI | | P |
| --- | --- | --- | --- | --- | --- | --- | --- | --- | --- | --- | --- |
|  |  |  |  |  |  |  | Beta | P | Beta | P |  |
| rs4074720 | 10 | 71 | chr10:114654837..114916586 | 261.75 | T | C | 0.0175 | 6.38E-03 | 0.2000 | 1.10E-150 | 6.66E-171 |
| rs234864 | 11 | 4 | chr11:2691471..2857297 | 165.827 | A | G | -0.0142 | 2.79E-02 | -0.1100 | 2.30E-44 | 7.75E-51 |
| rs76550717 | 11 | 14 | chr11:65560785..72477815 | 6917.03 | G | A | -0.0200 | 2.14E-02 | -0.1300 | 3.10E-35 | 1.65E-41 |
| rs1046317 | 4 | 110 | chr4:6263996..6319089 | 55.094 | C | T | -0.0172 | 1.15E-02 | 0.0950 | 1.90E-32 | 6.13E-35 |
| rs11712037 | 3 | 94 | chr3:12027240..12499264 | 472.025 | G | C | -0.0234 | 1.71E-02 | -0.1200 | 6.50E-25 | 2.58E-30 |
| rs703967 | 10 | 125 | chr10:71332301..81018948 | 9686.65 | C | A | 0.0169 | 8.17E-03 | -0.0800 | 3.00E-26 | 5.89E-29 |
| rs74643044 | 5 | 15 | chr5:101123995..102726073 | 1602.08 | C | T | 0.0308 | 2.97E-02 | 0.1800 | 4.40E-25 | 2.47E-28 |
| rs1215470 | 13 | 4 | chr13:80656382..80706455 | 50.074 | C | T | -0.0142 | 4.39E-02 | -0.0880 | 3.80E-26 | 4.20E-28 |
| rs849336 | 7 | 3 | chr7:28223990..28256240 | 32.251 | G | A | 0.0139 | 3.80E-02 | -0.0830 | 2.20E-26 | 7.04E-28 |
| rs2258238 | 12 | 158 | chr12:66164800..66379504 | 214.705 | T | A | -0.0403 | 1.06E-04 | 0.1200 | 1.90E-21 | 8.50E-28 |
| rs9379084 | 6 | 12 | chr6:7118990..7267313 | 148.324 | A | G | -0.0218 | 3.38E-02 | -0.1200 | 3.80E-23 | 1.80E-25 |
| rs75265117 | 2 | 42 | chr2:165502911..165675693 | 172.783 | G | C | 0.0236 | 1.59E-02 | -0.1100 | 1.10E-21 | 1.99E-25 |
| rs10741452 | 11 | 5 | chr11:92678397..92701596 | 23.2 | G | A | -0.0132 | 4.88E-02 | 0.0790 | 1.00E-23 | 9.85E-25 |
| rs340882 | 1 | 41 | chr1:205044339..219731273 | 14686.9 | G | C | -0.0134 | 4.15E-02 | 0.0710 | 2.30E-20 | 1.86E-21 |
| rs1490384 | 6 | 210 | chr6:126623947..127167072 | 543.126 | T | C | 0.0324 | 3.42E-07 | 0.0560 | 2.50E-14 | 5.83E-20 |
| rs9350294 | 6 | 19 | chr6:20639604..26336696 | 5697.09 | C | T | 0.0153 | 2.14E-02 | 0.0670 | 7.00E-18 | 1.03E-19 |
| rs79111706 | 2 | 20 | chr2:43620587..44020441 | 399.855 | C | T | -0.0228 | 4.95E-02 | -0.1200 | 4.50E-17 | 4.02E-19 |
| rs601945 | 6 | 181 | chr6:31092767..32636434 | 1543.67 | G | A | -0.0183 | 2.39E-02 | 0.0850 | 1.50E-17 | 7.53E-19 |
| rs9922619 | 16 | 98 | chr16:53798523..53845487 | 46.965 | T | G | 0.0272 | 2.21E-05 | 0.0540 | 6.00E-13 | 3.05E-17 |
| rs3768321 | 1 | 99 | chr1:39551488..40073011 | 521.524 | T | G | -0.0199 | 1.30E-02 | 0.0750 | 9.60E-16 | 3.70E-17 |

Chr: chromosome; Pos: chromosome position; Kb: the length of the shared genomic region; N: number of identified significant SNPs in this region; A1: effect allele; A2: another allele; Beta: effect size in single-phenotype genome-wide association study; P: p value.

**Supplementary Table 12. Shared genetic loci of choice for ground coffee and body mass index (BMI) identified by cross-phenotype association analysis (top 20).**

| Index SNP | Chr | N | Pos | Kb | A1 | A2 | ground coffee | | BMI | | P |
| --- | --- | --- | --- | --- | --- | --- | --- | --- | --- | --- | --- |
|  |  |  |  |  |  |  | Beta | P | Beta | P |  |
| rs8047395 | 16 | 65 | chr16:53614746..53912466 | 297.721 | A | G | 0.0266 | 2.92E-05 | 0.0642 | 0.00E+00 | 0.00E+00 |
| rs10182181 | 2 | 104 | chr2:24692809..29093803 | 4401 | G | A | -0.0151 | 1.74E-02 | 0.0325 | 6.70E-90 | 2.10E-98 |
| rs11030104 | 11 | 73 | chr11:18363569..30378559 | 12015 | G | A | -0.0164 | 3.84E-02 | -0.0393 | 2.00E-83 | 3.92E-92 |
| rs7531118 | 1 | 225 | chr1:65896308..77981099 | 12084.8 | C | T | 0.0438 | 8.57E-12 | 0.0256 | 3.60E-54 | 3.21E-79 |
| rs8049439 | 16 | 124 | chr16:24609208..29001460 | 4392.25 | C | T | -0.0500 | 1.16E-14 | 0.0265 | 1.50E-58 | 9.75E-74 |
| rs2307111 | 5 | 129 | chr5:74299144..81152078 | 6852.94 | C | T | 0.0196 | 2.62E-03 | -0.0265 | 1.60E-58 | 1.19E-65 |
| rs11713193 | 3 | 599 | chr3:41260017..56239203 | 14979.2 | A | G | -0.0430 | 1.30E-11 | 0.0239 | 2.40E-44 | 7.46E-54 |
| rs13107325 | 4 | 79 | chr4:94407841..105009833 | 10602 | T | C | -0.0526 | 1.43E-05 | 0.0470 | 1.10E-47 | 7.73E-52 |
| rs1412235 | 9 | 27 | chr9:23343995..36993788 | 13649.8 | C | G | -0.0171 | 1.16E-02 | 0.0246 | 6.00E-45 | 2.83E-50 |
| rs2820311 | 1 | 25 | chr1:201760981..201887382 | 126.402 | G | A | -0.0173 | 1.04E-02 | 0.0235 | 4.10E-38 | 4.75E-41 |
| rs6932930 | 6 | 147 | chr6:25772047..43352898 | 17580.9 | A | G | -0.0197 | 9.92E-03 | -0.0242 | 1.60E-35 | 9.03E-41 |
| rs879620 | 16 | 26 | chr16:3575270..7136601 | 3561.33 | T | C | -0.0168 | 1.03E-02 | 0.0231 | 5.30E-38 | 1.09E-39 |
| rs3814424 | 5 | 45 | chr5:86584820..92356778 | 5771.96 | T | C | 0.0231 | 8.21E-03 | 0.0300 | 8.60E-35 | 1.19E-39 |
| rs6444106 | 3 | 45 | chr3:180373531..194839584 | 14466.1 | A | G | -0.0160 | 4.92E-02 | -0.0269 | 7.40E-39 | 1.53E-39 |
| rs12964689 | 18 | 105 | chr18:20913803..23124725 | 2210.92 | G | A | 0.0335 | 1.38E-07 | -0.0203 | 5.10E-32 | 4.92E-38 |
| rs4898530 | 12 | 45 | chr12:41962908..56491880 | 14529 | G | A | 0.0170 | 9.44E-03 | 0.0205 | 2.00E-34 | 4.35E-37 |
| rs2122042 | 3 | 478 | chr3:78608348..89778009 | 11169.7 | T | G | 0.0206 | 8.34E-03 | 0.0235 | 2.30E-31 | 1.42E-35 |
| rs1167827 | 7 | 43 | chr7:70010479..78162105 | 8151.63 | G | A | -0.0141 | 2.83E-02 | 0.0203 | 2.80E-34 | 1.87E-34 |
| rs4237643 | 11 | 59 | chr11:43599791..48333360 | 4733.57 | G | T | 0.0167 | 1.54E-02 | -0.0223 | 4.30E-33 | 2.62E-33 |
| rs12369179 | 12 | 29 | chr12:114562870..132219427 | 17656.6 | T | C | -0.0218 | 4.93E-02 | -0.0359 | 2.50E-31 | 9.91E-33 |

Chr: chromosome; Pos: chromosome position; Kb: the length of the shared genomic region; N: number of identified significant SNPs in this region; A1: effect allele; A2: another allele; Beta: effect size in single-phenotype genome-wide association study; P: p value.

**Supplementary Table 13. Shared genetic loci of choice for ground coffee and fasting glucose (FG) identified by cross-phenotype association analysis (top 20).**

| Index SNP | Chr | N | Pos | Kb | A1 | A2 | ground coffee | | FG | | P |
| --- | --- | --- | --- | --- | --- | --- | --- | --- | --- | --- | --- |
|  |  |  |  |  |  |  | Beta | P | Beta | P |  |
| rs563694 | 2 | 26 | chr2:169753415..169802252 | 48.838 | A | C | -0.0138 | 3.86E-02 | 0.0690 | 1.25E-71 | 1.33E-81 |
| rs4753426 | 11 | 1 | chr11:92701596..92701596 | 0.001 | C | T | -0.0125 | 4.95E-02 | 0.0480 | 5.34E-34 | 6.46E-38 |
| rs12325113 | 16 | 33 | chr16:28490517..28992646 | 502.13 | C | T | -0.0514 | 2.30E-15 | 0.0075 | 4.29E-02 | 9.69E-17 |
| rs10822182 | 10 | 22 | chr10:65124098..65400080 | 275.983 | A | G | 0.0420 | 4.30E-11 | -0.0074 | 4.33E-02 | 5.84E-12 |
| rs4339469 | 6 | 9 | chr6:98310291..98491210 | 180.92 | G | T | -0.0415 | 2.94E-10 | 0.0077 | 4.95E-02 | 5.38E-11 |
| rs1378942 | 15 | 1 | chr15:75077367..75077367 | 0.001 | A | C | 0.0424 | 5.26E-10 | -0.0075 | 4.97E-02 | 1.03E-10 |
| rs26950 | 5 | 3 | chr5:59823118..60127724 | 304.607 | T | C | 0.0397 | 9.36E-10 | -0.0088 | 2.05E-02 | 1.51E-10 |
| rs2225899 | 14 | 14 | chr14:30122409..30177166 | 54.758 | T | C | -0.0392 | 1.58E-09 | 0.0089 | 1.68E-02 | 2.56E-10 |
| rs10207150 | 2 | 28 | chr2:126022880..126329604 | 306.725 | C | T | -0.0566 | 1.31E-08 | -0.0130 | 2.91E-02 | 1.28E-09 |
| rs332821 | 1 | 2 | chr1:61674754..61678557 | 3.804 | A | T | 0.0372 | 6.59E-09 | -0.0084 | 3.88E-02 | 1.66E-09 |
| rs174548 | 11 | 18 | chr11:61551356..61609750 | 58.395 | G | C | 0.0251 | 2.37E-04 | -0.0230 | 5.31E-09 | 2.67E-09 |
| rs6927072 | 6 | 3 | chr6:152251080..152263360 | 12.281 | G | T | 0.0380 | 1.21E-08 | -0.0090 | 2.48E-02 | 2.84E-09 |
| rs1788817 | 18 | 24 | chr18:21075441..21161134 | 85.694 | G | A | 0.0348 | 4.46E-08 | -0.0084 | 2.10E-02 | 1.21E-08 |
| rs197377 | 1 | 1 | chr1:112291644..112291644 | 0.001 | T | C | -0.0429 | 8.42E-08 | 0.0160 | 3.92E-02 | 2.77E-08 |
| rs1454909 | 5 | 4 | chr5:57244956..57309750 | 64.795 | T | C | -0.0330 | 3.13E-07 | -0.0090 | 1.62E-02 | 3.90E-08 |

Chr: chromosome; Pos: chromosome position; Kb: the length of the shared genomic region; N: number of identified significant SNPs in this region; A1: effect allele; A2: another allele; Beta: effect size in single-phenotype genome-wide association study; P: p value.

**Supplementary Table 14. Shared genetic loci of choice for ground coffee and fasting insulin (FI) identified by cross-phenotype association analysis (top 20).**

| Index SNP | Chr | N | Pos | Kb | A1 | A2 | ground coffee | | FI | | P |
| --- | --- | --- | --- | --- | --- | --- | --- | --- | --- | --- | --- |
|  |  |  |  |  |  |  | Beta | P | Beta | P |  |
| rs12325113 | 16 | 47 | chr16:28490517..28969480 | 478.964 | C | T | -0.0514 | 2.30E-15 | 0.0130 | 1.30E-03 | 4.42E-17 |
| rs2393986 | 10 | 65 | chr10:64904071..65400080 | 496.01 | T | A | 0.0424 | 3.00E-11 | -0.0083 | 3.00E-02 | 4.35E-12 |
| rs2347867 | 6 | 9 | chr6:152225383..152263360 | 37.978 | A | G | 0.0407 | 7.52E-10 | -0.0100 | 1.21E-02 | 1.41E-10 |
| rs26950 | 5 | 1 | chr5:59823118..59823118 | 0.001 | T | C | 0.0397 | 9.36E-10 | -0.0083 | 3.61E-02 | 2.14E-10 |
| rs2225899 | 14 | 14 | chr14:30122409..30177166 | 54.758 | T | C | -0.0392 | 1.58E-09 | 0.0089 | 2.19E-02 | 3.56E-10 |
| rs2271960 | 3 | 24 | chr3:49744890..49890613 | 145.724 | C | T | -0.0369 | 6.63E-09 | 0.0089 | 1.97E-02 | 1.72E-09 |
| rs11960699 | 5 | 12 | chr5:57244956..57309750 | 64.795 | A | C | -0.0332 | 2.36E-07 | -0.0160 | 6.65E-05 | 6.58E-09 |

Chr: chromosome; Pos: chromosome position; Kb: the length of the shared genomic region; N: number of identified significant SNPs in this region; A1: effect allele; A2: another allele; Beta: effect size in single-phenotype genome-wide association study; P: p value.

**Supplementary Table 15. Shared genetic loci of choice for ground coffee and insulin resistance (HOMA-IR) identified by cross-phenotype association analysis (top 20).**

| Index SNP | Chr | N | Pos | Kb | A1 | A2 | ground coffee | | HOMA-IR | | P |
| --- | --- | --- | --- | --- | --- | --- | --- | --- | --- | --- | --- |
|  |  |  |  |  |  |  | Beta | P | Beta | P |  |
| rs12325113 | 16 | 41 | chr16:28490517..28969480 | 478.964 | C | T | -0.0514 | 2.30E-15 | 0.0120 | 3.24E-03 | 6.66E-17 |
| rs2681780 | 3 | 35 | chr3:49744890..50027774 | 282.885 | T | C | -0.0437 | 6.32E-12 | 0.0082 | 3.90E-02 | 8.93E-13 |
| rs2393986 | 10 | 67 | chr10:64904071..65400080 | 496.01 | T | A | 0.0424 | 3.00E-11 | -0.0100 | 9.87E-03 | 4.05E-12 |
| rs2347867 | 6 | 9 | chr6:152225383..152263360 | 37.978 | A | G | 0.0407 | 7.52E-10 | -0.0098 | 2.05E-02 | 1.66E-10 |
| rs26950 | 5 | 3 | chr5:59805467..59825300 | 19.834 | T | C | 0.0397 | 9.36E-10 | -0.0110 | 9.06E-03 | 1.78E-10 |
| rs2225899 | 14 | 9 | chr14:30145432..30177166 | 31.735 | T | C | -0.0392 | 1.58E-09 | 0.0082 | 4.28E-02 | 4.50E-10 |
| rs303753 | 18 | 11 | chr18:21074922..21154024 | 79.103 | A | G | -0.0398 | 3.44E-09 | 0.0085 | 4.44E-02 | 1.06E-09 |
| rs332821 | 1 | 2 | chr1:61674754..61678557 | 3.804 | A | T | 0.0372 | 6.59E-09 | -0.0091 | 3.99E-02 | 2.16E-09 |
| rs7581261 | 2 | 1 | chr2:126229399..126229399 | 0.001 | C | A | -0.0548 | 3.00E-08 | -0.0130 | 4.92E-02 | 3.97E-09 |
| rs11960699 | 5 | 5 | chr5:57244956..57309750 | 64.795 | A | C | -0.0332 | 2.36E-07 | -0.0150 | 3.16E-04 | 9.22E-09 |

Chr: chromosome; Pos: chromosome position; Kb: the length of the shared genomic region; N: number of identified significant SNPs in this region; A1: effect allele; A2: another allele; Beta: effect size in single-phenotype genome-wide association study; P: p value.

**Supplementary Table 16. Shared genetic loci of choice for instant coffee and type 2 diabetes mellitus (T2DM) identified by cross-phenotype association analysis (top 20).**

| Index SNP | Chr | N | Pos | Kb | A1 | A2 | instant coffee | | T2DM | | P |
| --- | --- | --- | --- | --- | --- | --- | --- | --- | --- | --- | --- |
|  |  |  |  |  |  |  | Beta | P | Beta | P |  |
| rs1421085 | 16 | 123 | chr16:53599637..53877592 | 277.956 | C | T | -0.0252 | 2.03E-06 | 0.1200 | 2.40E-78 | 6.73E-90 |
| rs10937719 | 4 | 65 | chr4:3312606..6315406 | 3002.8 | G | C | 0.0116 | 3.00E-02 | 0.0870 | 2.90E-40 | 2.93E-43 |
| rs13388242 | 2 | 3 | chr2:227020521..227022036 | 1.516 | A | G | 0.0123 | 2.57E-02 | -0.0810 | 6.30E-33 | 5.27E-36 |
| rs11708067 | 3 | 57 | chr3:123049938..123137791 | 87.854 | G | A | 0.0134 | 2.73E-02 | -0.0890 | 1.30E-31 | 6.39E-34 |
| rs76550717 | 11 | 14 | chr11:65390554..72708204 | 7317.65 | G | A | 0.0154 | 3.06E-02 | -0.1000 | 6.20E-32 | 1.06E-32 |
| rs703977 | 10 | 133 | chr10:71457566..81018948 | 9561.38 | G | T | -0.0190 | 2.83E-04 | -0.0710 | 5.00E-28 | 1.64E-32 |
| rs849335 | 7 | 5 | chr7:23512896..28232457 | 4719.56 | C | T | -0.0112 | 4.24E-02 | -0.0760 | 2.20E-30 | 3.29E-32 |
| rs113397277 | 10 | 29 | chr10:114596674..114674436 | 77.763 | C | T | -0.0315 | 2.68E-02 | 0.1800 | 6.00E-26 | 2.84E-28 |
| rs2262889 | 12 | 134 | chr12:66162603..66357946 | 195.344 | A | G | 0.0313 | 3.94E-04 | 0.1100 | 1.70E-23 | 5.46E-27 |
| rs2796441 | 9 | 38 | chr9:81364782..84384450 | 3019.67 | A | G | -0.0109 | 3.89E-02 | -0.0660 | 8.50E-24 | 2.29E-25 |
| rs1912980 | 2 | 27 | chr2:60561692..60599100 | 37.409 | T | C | 0.0134 | 1.09E-02 | -0.0560 | 4.60E-18 | 1.13E-20 |
| rs12367102 | 12 | 120 | chr12:26440698..33407093 | 6966.4 | T | C | 0.0133 | 3.27E-02 | -0.0660 | 7.00E-18 | 9.35E-20 |
| rs1377807 | 17 | 158 | chr17:3880546..4281407 | 400.862 | C | G | -0.0163 | 3.71E-03 | 0.0570 | 5.70E-17 | 9.56E-20 |
| rs429358 | 19 | 12 | chr19:45387459..45415935 | 28.477 | C | T | -0.0158 | 2.83E-02 | -0.0800 | 1.80E-18 | 2.30E-19 |
| rs111503677 | 13 | 4 | chr13:80617309..80758943 | 141.635 | G | A | -0.0132 | 4.70E-02 | -0.0720 | 3.30E-18 | 5.26E-19 |
| rs2307111 | 5 | 59 | chr5:74357475..78602863 | 4245.39 | C | T | -0.0133 | 1.30E-02 | -0.0530 | 3.30E-16 | 1.16E-17 |
| rs10974438 | 9 | 26 | chr9:4263055..4298955 | 35.901 | C | A | -0.0171 | 1.78E-03 | 0.0510 | 1.60E-14 | 1.51E-17 |
| rs9267658 | 6 | 60 | chr6:31320242..32627747 | 1307.51 | C | T | 0.0224 | 2.32E-03 | 0.0720 | 1.20E-15 | 1.78E-17 |
| rs117564240 | 10 | 2 | chr10:114764619..114787163 | 22.545 | G | T | -0.0307 | 2.11E-02 | -0.1300 | 4.90E-16 | 2.51E-17 |
| rs12248718 | 10 | 3 | chr10:12242326..12293699 | 51.374 | G | A | -0.0147 | 1.07E-02 | 0.0530 | 1.90E-14 | 1.51E-16 |

Chr: chromosome; Pos: chromosome position; Kb: the length of the shared genomic region; N: number of identified significant SNPs in this region; A1: effect allele; A2: another allele; Beta: effect size in single-phenotype genome-wide association study; P: p value.

**Supplementary Table 17. Shared genetic loci of choice for instant coffee and body mass index (BMI) identified by cross-phenotype association analysis (top 20).**

| Index SNP | Chr | N | Pos | Kb | A1 | A2 | instant coffee | | BMI | | P |
| --- | --- | --- | --- | --- | --- | --- | --- | --- | --- | --- | --- |
|  |  |  |  |  |  |  | Beta | P | Beta | P |  |
| rs8047395 | 16 | 110 | chr16:50986308..53912466 | 2926.16 | A | G | -0.0193 | 2.19E-04 | 0.0642 | 0.00E+00 | 0.00E+00 |
| rs953442 | 18 | 14 | chr18:53210302..58177883 | 4967.58 | C | T | 0.0124 | 4.04E-02 | 0.0491 | 3.40E-147 | 1.26E-157 |
| rs943005 | 6 | 38 | chr6:50798526..54117567 | 3319.04 | T | C | -0.0149 | 3.19E-02 | 0.0415 | 2.70E-83 | 1.81E-92 |
| rs7531118 | 1 | 212 | chr1:72423184..73251013 | 827.83 | C | T | -0.0357 | 1.15E-11 | 0.0256 | 3.60E-54 | 1.88E-75 |
| rs7127507 | 11 | 24 | chr11:27419134..32160524 | 4741.39 | C | T | 0.0121 | 2.94E-02 | 0.0287 | 3.10E-62 | 1.12E-67 |
| rs2307111 | 5 | 104 | chr5:74276566..75015242 | 738.677 | C | T | -0.0133 | 1.30E-02 | -0.0265 | 1.60E-58 | 3.66E-65 |
| rs8049439 | 16 | 81 | chr16:24615813..30041966 | 5426.15 | C | T | 0.0207 | 1.02E-04 | 0.0265 | 1.50E-58 | 3.66E-65 |
| rs13107325 | 4 | 17 | chr4:94407841..105009833 | 10602 | T | C | -0.0337 | 7.05E-04 | 0.0470 | 1.10E-47 | 1.69E-53 |
| rs7647305 | 3 | 3 | chr3:185470700..185834290 | 363.591 | C | T | -0.0134 | 3.59E-02 | 0.0298 | 9.90E-50 | 7.15E-53 |
| rs11713193 | 3 | 455 | chr3:41225545..51728573 | 10503 | A | G | 0.0187 | 3.36E-04 | 0.0239 | 2.40E-44 | 3.78E-47 |
| rs879620 | 16 | 29 | chr16:414930..7136601 | 6721.67 | T | C | 0.0126 | 1.89E-02 | 0.0231 | 5.30E-38 | 2.09E-39 |
| rs3814424 | 5 | 65 | chr5:86605943..95470989 | 8865.05 | T | C | -0.0158 | 2.71E-02 | 0.0300 | 8.60E-35 | 2.02E-37 |
| rs12964689 | 18 | 54 | chr18:20985632..23090640 | 2105.01 | G | A | -0.0184 | 4.22E-04 | -0.0203 | 5.10E-32 | 6.67E-35 |
| rs4857329 | 3 | 224 | chr3:84145071..94249591 | 10104.5 | G | A | -0.0117 | 2.51E-02 | -0.0202 | 5.30E-32 | 6.97E-34 |
| rs2278483 | 2 | 38 | chr2:24725464..25070645 | 345.182 | T | C | -0.0197 | 1.67E-03 | -0.0215 | 5.40E-29 | 5.23E-31 |
| rs7832708 | 8 | 1106 | chr8:4298057..15207175 | 10909.1 | T | C | -0.0167 | 1.39E-03 | 0.0168 | 1.30E-24 | 1.28E-29 |
| rs4788199 | 16 | 4 | chr16:29970857..30008644 | 37.788 | G | C | -0.0111 | 3.34E-02 | 0.0180 | 2.10E-25 | 2.25E-27 |
| rs6857 | 19 | 8 | chr19:45392254..45802863 | 410.61 | T | C | -0.0138 | 4.68E-02 | -0.0244 | 2.60E-25 | 3.74E-27 |
| rs11866219 | 16 | 31 | chr16:69549749..71442416 | 1892.67 | C | A | -0.0113 | 3.57E-02 | -0.0184 | 3.10E-25 | 2.91E-25 |
| rs10128597 | 11 | 7 | chr11:838722..8694830 | 7856.11 | A | G | -0.0116 | 4.90E-02 | -0.0190 | 3.90E-24 | 3.31E-24 |

Chr: chromosome; Pos: chromosome position; Kb: the length of the shared genomic region; N: number of identified significant SNPs in this region; A1: effect allele; A2: another allele; Beta: effect size in single-phenotype genome-wide association study; P: p value.

**Supplementary Table 18. Shared genetic loci of choice for instant coffee and fasting glucose (FG) identified by cross-phenotype association analysis (top 20).**

| Index SNP | Chr | N | Pos | Kb | A1 | A2 | instant coffee | | FG | | P |
| --- | --- | --- | --- | --- | --- | --- | --- | --- | --- | --- | --- |
|  |  |  |  |  |  |  | Beta | P | Beta | P |  |
| rs10278336 | 7 | 2 | chr7:44245363..44247258 | 1.896 | G | A | -0.0105 | 4.73E-02 | -0.0360 | 1.90E-20 | 8.66E-23 |
| rs10497348 | 2 | 6 | chr2:169718301..169771196 | 52.896 | A | G | 0.0260 | 4.75E-02 | 0.0670 | 9.83E-13 | 3.12E-13 |
| rs11717195 | 3 | 3 | chr3:123065778..123093530 | 27.753 | C | T | 0.0133 | 2.82E-02 | -0.0290 | 1.11E-09 | 4.16E-10 |
| rs10822182 | 10 | 5 | chr10:65124098..65353755 | 229.658 | A | G | -0.0307 | 4.20E-09 | -0.0074 | 4.33E-02 | 9.00E-10 |

Chr: chromosome; Pos: chromosome position; Kb: the length of the shared genomic region; N: number of identified significant SNPs in this region; A1: effect allele; A2: another allele; Beta: effect size in single-phenotype genome-wide association study; P: p value.

**Supplementary Table 19. Shared genetic loci of choice for instant coffee and fasting insulin (FI) identified by cross-phenotype association analysis (top 20).**

| Index SNP | Chr | N | Pos | Kb | A1 | A2 | instant coffee | | FI | | P |
| --- | --- | --- | --- | --- | --- | --- | --- | --- | --- | --- | --- |
|  |  |  |  |  |  |  | Beta | P | Beta | P |  |
| rs2393986 | 10 | 48 | chr10:64904071..65353755 | 449.685 | T | A | -0.0311 | 2.69E-09 | -0.0083 | 3.00E-02 | 6.09E-10 |

Chr: chromosome; Pos: chromosome position; Kb: the length of the shared genomic region; N: number of identified significant SNPs in this region; A1: effect allele; A2: another allele; Beta: effect size in single-phenotype genome-wide association study; P: p value.

**Supplementary Table 20. Shared genetic loci of choice for instant coffee and insulin resistance (HOMA-IR) identified by cross-phenotype association analysis (top 20).**

| Index SNP | Chr | N | Pos | Kb | A1 | A2 | instant coffee | | HOMA-IR | | P |
| --- | --- | --- | --- | --- | --- | --- | --- | --- | --- | --- | --- |
|  |  |  |  |  |  |  | Beta | P | Beta | P |  |
| rs2393986 | 10 | 50 | chr10:64904071..65353755 | 449.685 | T | A | -0.0311 | 2.69E-09 | -0.0100 | 9.87E-03 | 5.50E-10 |

Chr: chromosome; Pos: chromosome position; Kb: the length of the shared genomic region; N: number of identified significant SNPs in this region; A1: effect allele; A2: another allele; Beta: effect size in single-phenotype genome-wide association study; P: p value.

**Supplementary Table 21. MR results of coffee intake and T2DM as well as T2DM-related phenotypes.**

| T2DM/related phenotypes | CAUSE | | IVW | | MR Egger regression | | Simple median | | Weighted median | | Penalized weighted median | | Simple mode | | Weighted mode | |
| --- | --- | --- | --- | --- | --- | --- | --- | --- | --- | --- | --- | --- | --- | --- | --- | --- |
|  | b | p | b | p | b | p | b | p | b | p | b | p | b | p | b | p |
| T2DM | 0.31 | 3.30E-02 | 1.00 | 1.11E-03 | 0.82 | 2.02E-01 | 0.84 | 1.58E-06 | 0.53 | 5.63E-06 | 0.52 | 2.33E-05 | 0.83 | 1.11E-03 | 0.56 | 2.45E-05 |
| T2DM (adjusted for BMI) | 0 | 8.40E-01 | 0.50 | 3.60E-03 | 0.60 | 9.77E-02 | 0.66 | 1.11E-04 | 0.35 | 1.33E-02 | 0.34 | 1.23E-02 | 0.75 | 2.33E-02 | 0.35 | 1.89E-02 |
| BMI | 0.35 | 1.80E-05 | 0.86 | 7.96E-03 | 0.13 | 8.23E-01 | 0.93 | 1.91E-08 | 0.26 | 1.75E-15 | 0.26 | 2.78E-15 | 0.55 | 5.80E-04 | 0.27 | 3.10E-07 |
| FG | 0 | 9.10E-01 | 0.06 | 4.10E-01 | 0.01 | 9.42E-01 | 0.02 | 8.65E-01 | 0.03 | 6.99E-01 | 0.03 | 7.25E-01 | 0.08 | 5.76E-01 | 0.04 | 5.87E-01 |
| FI | 0 | 6.20E-01 | 0.20 | 1.94E-02 | 0.11 | 5.16E-01 | 0.10 | 4.20E-01 | 0.12 | 2.15E-01 | 0.10 | 2.48E-01 | 0.04 | 8.56E-01 | 0.09 | 3.16E-01 |
| HOMA-IR | 0 | 1.90E-01 | 0.21 | 1.55E-02 | 0.10 | 5.34E-01 | 0.09 | 4.87E-01 | 0.09 | 3.09E-01 | 0.09 | 2.99E-01 | 0.01 | 9.78E-01 | 0.08 | 3.84E-01 |
| HOMA-β | 0 | 6.20E-02 | 0.16 | 3.35E-03 | 0.14 | 1.98E-01 | 0.12 | 2.18E-01 | 0.11 | 1.17E-01 | 0.11 | 1.19E-01 | 0.12 | 3.41E-01 | 0.11 | 1.54E-01 |

**Supplementary Table 22 MR results of choice of decaffeinated coffee and T2DM as well as T2DM-related phenotypes.**

| T2DM/related phenotypes | CAUSE | | IVW | | MR Egger regression | | Simple median | | Weighted median | | Penalized weighted median | | Simple mode | | Weighted mode | |
| --- | --- | --- | --- | --- | --- | --- | --- | --- | --- | --- | --- | --- | --- | --- | --- | --- |
|  | b | p | b | p | b | p | b | p | b | p | b | p | b | p | b | p |
| T2DM | 0.29 | 2.30E-02 | 0.07 | 2.74E-01 | 0.13 | 4.05E-01 | 0.05 | 5.14E-01 | 0.05 | 4.47E-01 | 0.04 | 5.37E-01 | -0.01 | 9.47E-01 | -0.01 | 9.53E-01 |
| T2DM (adjusted for BMI) | 0 | 5.10E-01 | 0.02 | 7.76E-01 | -0.02 | 8.66E-01 | -0.01 | 8.79E-01 | 0.00 | 9.63E-01 | 0.00 | 9.94E-01 | -0.07 | 5.94E-01 | -0.03 | 7.71E-01 |
| BMI | 0 | 2.80E-01 | 0.11 | 3.41E-02 | -0.32 | 8.27E-01 | 0.13 | 2.43E-03 | 0.13 | 4.74E-03 | 0.14 | 5.15E-03 | 0.14 | 1.06E-01 | 0.14 | 1.85E-01 |
| FG | 0 | 1.00E+00 | 0.04 | 3.78E-01 | -0.08 | 5.48E-01 | 0.03 | 5.80E-01 | 0.02 | 7.65E-01 | 0.02 | 7.71E-01 | 0.02 | 8.66E-01 | -0.01 | 9.46E-01 |
| FI | 0 | 5.90E-01 | 0.00 | 9.87E-01 | 0.12 | 4.94E-01 | -0.09 | 1.76E-01 | -0.06 | 3.60E-01 | -0.08 | 2.32E-01 | -0.11 | 3.62E-01 | -0.10 | 2.96E-01 |
| HOMA-IR | 0 | 9.40E-01 | 0.01 | 8.18E-01 | 0.08 | 5.68E-01 | -0.04 | 5.73E-01 | -0.03 | 6.94E-01 | -0.03 | 6.97E-01 | -0.06 | 5.53E-01 | -0.05 | 5.59E-01 |
| HOMA-β | 0 | 9.90E-01 | 0.03 | 5.47E-01 | 0.12 | 4.36E-01 | 0.03 | 6.16E-01 | 0.06 | 3.51E-01 | 0.06 | 3.38E-01 | 0.03 | 7.37E-01 | 0.05 | 4.91E-01 |

**Supplementary Table 23. MR results of choice of ground coffee and T2DM as well as T2DM-related phenotypes.**

| T2DM/related phenotypes | CAUSE | | IVW | | MR Egger regression | | Simple median | | Weighted median | | Penalized weighted median | | Simple mode | | Weighted mode | |
| --- | --- | --- | --- | --- | --- | --- | --- | --- | --- | --- | --- | --- | --- | --- | --- | --- |
|  | b | p | b | p | b | p | b | p | b | p | b | p | b | p | b | p |
| T2DM | -0.2 | 4.70E-10 | -0.06 | 6.20E-01 | 0.04 | 9.58E-01 | -0.17 | 1.68E-02 | -0.17 | 1.66E-02 | -0.33 | 2.76E-07 | -0.32 | 1.38E-03 | -0.32 | 7.41E-04 |
| T2DM (adjusted for BMI) | -0.11 | 4.60E-05 | -0.04 | 6.60E-01 | -0.39 | 5.51E-01 | -0.15 | 3.33E-02 | -0.14 | 3.37E-02 | -0.17 | 8.66E-03 | -0.16 | 1.09E-01 | -0.16 | 1.59E-01 |
| BMI | -0.08 | 6.50E-05 | 0.03 | 6.14E-01 | 0.26 | 5.62E-01 | -0.02 | 3.86E-01 | -0.02 | 3.49E-01 | -0.04 | 7.32E-02 | -0.04 | 1.23E-01 | -0.03 | 1.85E-01 |
| FG | -0.03 | 4.60E-02 | -0.04 | 2.06E-01 | 0.24 | 2.28E-01 | -0.04 | 3.03E-01 | -0.03 | 3.72E-01 | -0.03 | 3.69E-01 | -0.05 | 5.29E-01 | -0.03 | 6.30E-01 |
| FI | 0 | 1.30E-01 | -0.03 | 2.47E-01 | 0.21 | 2.54E-01 | -0.02 | 5.63E-01 | -0.02 | 5.75E-01 | -0.02 | 5.81E-01 | -0.02 | 7.40E-01 | -0.02 | 7.80E-01 |
| HOMA-IR | 0 | 2.40E-01 | -0.04 | 1.53E-01 | 0.26 | 1.95E-01 | -0.07 | 8.99E-02 | -0.07 | 1.12E-01 | -0.07 | 1.04E-01 | -0.07 | 4.07E-01 | -0.07 | 4.18E-01 |
| HOMA-β | 0 | 9.40E-01 | -0.02 | 4.90E-01 | 0.11 | 5.16E-01 | -0.03 | 3.99E-01 | -0.03 | 4.15E-01 | -0.03 | 4.16E-01 | -0.04 | 5.12E-01 | -0.04 | 5.10E-01 |

**Supplementary Table 24. MR results of choice of instant coffee and T2DM as well as T2DM-related phenotypes.**

| T2DM/related phenotypes | CAUSE | | IVW | | MR Egger regression | | Simple median | | Weighted median | | Penalized weighted median | | Simple mode | | Weighted mode | |
| --- | --- | --- | --- | --- | --- | --- | --- | --- | --- | --- | --- | --- | --- | --- | --- | --- |
|  | b | p | b | p | b | p | b | p | b | p | b | p | b | p | b | p |
| T2DM | 0.17 | 2.90E-02 | -0.09 | 7.64E-01 | -1.18 | 7.36E-01 | -0.32 | 9.42E-02 | -0.40 | 1.47E-02 | -0.66 | 7.68E-06 | -0.57 | 2.95E-02 | -0.58 | 1.49E-02 |
| T2DM (adjusted for BMI) | 0 | 2.60E-01 | 0.04 | 8.67E-01 | 1.28 | 5.87E-01 | -0.05 | 7.45E-01 | -0.04 | 7.93E-01 | -0.10 | 5.11E-01 | -0.09 | 6.59E-01 | -0.05 | 7.64E-01 |
| BMI | 0 | 5.80E-02 | -0.18 | 3.43E-01 | -2.20 | 2.52E-01 | 0.01 | 8.67E-01 | 0.03 | 5.60E-01 | 0.03 | 5.04E-01 | 0.04 | 6.28E-01 | 0.05 | 3.89E-01 |
| FG | 0 | 4.50E-01 | 0.06 | 2.46E-01 | 0.04 | 9.40E-01 | 0.03 | 6.62E-01 | 0.06 | 4.02E-01 | 0.06 | 3.73E-01 | -0.01 | 9.37E-01 | 0.05 | 6.25E-01 |
| FI | 0 | 2.10E-01 | 0.02 | 7.33E-01 | -0.39 | 5.20E-01 | 0.00 | 9.71E-01 | 0.00 | 9.69E-01 | 0.00 | 9.69E-01 | -0.05 | 6.93E-01 | -0.05 | 6.65E-01 |
| HOMA-IR | 0 | 6.10E-01 | 0.02 | 7.69E-01 | -0.45 | 5.87E-01 | 0.02 | 8.40E-01 | 0.01 | 9.40E-01 | 0.01 | 9.40E-01 | -0.01 | 9.14E-01 | -0.04 | 7.62E-01 |
| HOMA-β | 0 | 4.50E-01 | -0.06 | 2.17E-01 | -0.24 | 6.78E-01 | -0.02 | 7.23E-01 | -0.03 | 5.77E-01 | -0.03 | 5.93E-01 | 0.01 | 9.08E-01 | 0.00 | 9.90E-01 |

**Supplementary Table 25. MR results of choice of other types coffee and T2DM as well as T2DM-related phenotypes.**

| T2DM/related phenotypes | CAUSE | | IVW | | MR Egger regression | | Simple median | | Weighted median | | Penalized weighted median | | Simple mode | | Weighted mode | |
| --- | --- | --- | --- | --- | --- | --- | --- | --- | --- | --- | --- | --- | --- | --- | --- | --- |
|  | b | p | b | p | b | p | b | p | b | p | b | p | b | p | b | p |
| T2DM | 0 | 1.00E+00 | 0.02 | 1.93E-01 | 0.06 | 1.80E-01 | 0.02 | 4.20E-01 | 0.02 | 4.27E-01 | 0.02 | 4.08E-01 | 0.02 | 7.18E-01 | 0.02 | 7.06E-01 |
| T2DM (adjusted for BMI) | 0 | 9.90E-01 | 0.02 | 3.26E-01 | 0.04 | 4.47E-01 | 0.03 | 2.70E-01 | 0.03 | 2.67E-01 | 0.03 | 2.71E-01 | 0.03 | 5.49E-01 | 0.04 | 5.07E-01 |
| BMI | 0 | 1.00E+00 | -0.02 | 8.84E-02 | 0.04 | 3.88E-01 | -0.01 | 2.80E-01 | -0.01 | 3.73E-01 | -0.01 | 4.44E-01 | -0.01 | 5.94E-01 | -0.01 | 6.23E-01 |
| FG | 0 | 1.00E+00 | 0.00 | 9.88E-01 | -0.06 | 5.96E-01 | 0.01 | 5.55E-01 | 0.01 | 5.96E-01 | 0.02 | 4.84E-01 | 0.02 | 6.58E-01 | 0.02 | 6.34E-01 |
| FI | 0 | 7.70E-01 | -0.01 | 6.62E-01 | -0.12 | 3.21E-01 | -0.01 | 6.63E-01 | -0.01 | 6.51E-01 | 0.00 | 9.36E-01 | 0.01 | 6.85E-01 | 0.01 | 8.16E-01 |
| HOMA-IR | 0 | 9.60E-01 | -0.02 | 5.35E-01 | -0.15 | 3.04E-01 | -0.01 | 7.48E-01 | -0.01 | 7.21E-01 | 0.00 | 9.14E-01 | 0.01 | 6.85E-01 | 0.01 | 8.30E-01 |
| HOMA-β | 0 | 8.50E-01 | -0.02 | 2.87E-01 | -0.11 | 1.24E-01 | 0.00 | 9.24E-01 | 0.00 | 9.25E-01 | 0.00 | 9.38E-01 | 0.00 | 9.84E-01 | 0.00 | 9.47E-01 |

**Supplementary Table 26. Heterogeneity test of Mendelian randomization studies for coffee intake.**

| outcome | method | Q | Q_df | Q_pval |
| --- | --- | --- | --- | --- |
| T2DM | Inverse variance weighted | 425.90 | 28 | 1.04E-72 |
|  | MR Egger | 424.03 | 27 | 6.24E-73 |
| T2DM(adjusted for BMI) | Inverse variance weighted | 97.51 | 28 | 1.28E-09 |
|  | MR Egger | 97.13 | 27 | 7.58E-10 |
| BMI | Inverse variance weighted | 2479.13 | 14 | 0.00E+00 |
|  | MR Egger | 2103.61 | 13 | 0.00E+00 |
| FG | Inverse variance weighted | 16.93 | 14 | 2.60E-01 |
|  | MR Egger | 16.71 | 13 | 2.13E-01 |
| FI | Inverse variance weighted | 24.90 | 14 | 3.55E-02 |
|  | MR Egger | 24.14 | 13 | 2.99E-02 |
| HOMA-IR | Inverse variance weighted | 22.92 | 14 | 6.17E-02 |
|  | MR Egger | 22.00 | 13 | 5.53E-02 |
| HOMA-β | Inverse variance weighted | 10.72 | 14 | 7.08E-01 |
|  | MR Egger | 10.65 | 13 | 6.40E-01 |

**Supplementary Table 27. Heterogeneity test of Mendelian randomization studies for decaffeinated coffee.**

| outcome | method | Q | Q_df | Q_pval |
| --- | --- | --- | --- | --- |
| T2DM | Inverse variance weighted | 6.09 | 1 | 1.36E-02 |
| T2DM (adjusted for BMI) | Inverse variance weighted | 0.30 | 1 | 5.84E-01 |
| BMI | MR Egger | 7.81 | 2 | 2.02E-02 |
|  | Inverse variance weighted | 8.23 | 3 | 4.14E-02 |

**Supplementary Table 28. Heterogeneity test of Mendelian randomization studies for ground coffee.**

| outcome | method | Q | Q_df | Q_pval |
| --- | --- | --- | --- | --- |
| T2DM | Inverse variance weighted | 182.02 | 18 | 3.82E-29 |
|  | MR Egger | 181.85 | 17 | 1.24E-29 |
| T2DM (adjusted for BMI) | Inverse variance weighted | 85.65 | 18 | 8.63E-11 |
|  | MR Egger | 84.15 | 17 | 6.96E-11 |
| BMI | Inverse variance weighted | 480.58 | 13 | 1.94E-94 |
|  | MR Egger | 469.84 | 12 | 5.77E-93 |
| FG | Inverse variance weighted | 17.95 | 13 | 1.60E-01 |
|  | MR Egger | 15.14 | 12 | 2.34E-01 |
| FI | Inverse variance weighted | 13.57 | 13 | 4.05E-01 |
|  | MR Egger | 11.62 | 12 | 4.76E-01 |
| HOMA-IR | Inverse variance weighted | 16.88 | 13 | 2.05E-01 |
|  | MR Egger | 13.84 | 12 | 3.11E-01 |
| HOMA-β | Inverse variance weighted | 15.52 | 13 | 2.76E-01 |
|  | MR Egger | 14.77 | 12 | 2.54E-01 |

**Supplementary Table 29. Heterogeneity test of Mendelian randomization studies for instant coffee.**

| outcome | method | Q | Q_df | Q_pval |
| --- | --- | --- | --- | --- |
| T2DM | Inverse variance weighted | 50.02 | 4 | 3.58E-10 |
|  | MR Egger | 48.14 | 3 | 1.99E-10 |
| T2DM (adjusted for BMI) | Inverse variance weighted | 17.32 | 4 | 1.67E-03 |
|  | MR Egger | 15.51 | 3 | 1.43E-03 |
| BMI | Inverse variance weighted | 264.69 | 4 | 4.45E-56 |
|  | MR Egger | 168.73 | 3 | 2.40E-36 |
| FG | Inverse variance weighted | 3.63 | 4 | 4.59E-01 |
|  | MR Egger | 3.62 | 3 | 3.05E-01 |
| FI | Inverse variance weighted | 4.28 | 4 | 3.69E-01 |
|  | MR Egger | 3.58 | 3 | 3.10E-01 |
| HOMA-IR | Inverse variance weighted | 7.17 | 4 | 1.27E-01 |
|  | MR Egger | 6.30 | 3 | 9.77E-02 |
| HOMA-β | Inverse variance weighted | 4.58 | 4 | 3.34E-01 |
|  | MR Egger | 4.41 | 3 | 2.21E-01 |

**Supplementary Table 30. Heterogeneity test of Mendelian randomization studies for other types of coffee.**

| outcome | method | Q | Q_df | Q_pval |
| --- | --- | --- | --- | --- |
| T2DM | Inverse variance weighted | 15.76 | 17 | 5.41E-01 |
|  | MR Egger | 14.88 | 16 | 5.34E-01 |
| T2DM (adjusted for BMI) | Inverse variance weighted | 14.24 | 17 | 6.50E-01 |
|  | MR Egger | 14.08 | 16 | 5.93E-01 |
| BMI | Inverse variance weighted | 15.70 | 6 | 1.55E-02 |
|  | MR Egger | 11.07 | 5 | 5.01E-02 |
| FG | Inverse variance weighted | 11.60 | 6 | 7.16E-02 |
|  | MR Egger | 10.86 | 5 | 5.42E-02 |
| FI | Inverse variance weighted | 14.70 | 6 | 2.27E-02 |
|  | MR Egger | 12.15 | 5 | 3.28E-02 |
| HOMA-IR | Inverse variance weighted | 17.50 | 6 | 7.60E-03 |
|  | MR Egger | 14.47 | 5 | 1.29E-02 |
| HOMA-β | Inverse variance weighted | 7.15 | 6 | 3.07E-01 |
|  | MR Egger | 4.54 | 5 | 4.74E-01 |

**Supplementary Table 31. Horizontal pleiotropy test of Mendelian randomization studies.**

| exposure | outcome | egger_intercept | se | pval |
| --- | --- | --- | --- | --- |
| coffee intake | T2DM | 0.0037 | 0.0109 | 0.7333 |
|  | T2DM (adjusted for BMI) | -0.0020 | 0.0061 | 0.7464 |
|  | BMI | 0.0172 | 0.0113 | 0.1516 |
|  | FG | 0.0010 | 0.0024 | 0.6866 |
|  | FI | 0.0019 | 0.0030 | 0.5321 |
|  | HOMA-IR | 0.0022 | 0.0030 | 0.4757 |
|  | HOMA-β | 0.0005 | 0.0019 | 0.7997 |
| Decaffeinated coffee | T2DM | NA | NA | NA |
|  | T2DM (adjusted for BMI) | NA | NA | NA |
|  | BMI | 0.0134 | 0.0404 | 0.7721 |
| Ground coffee | T2DM | -0.0044 | 0.0348 | 0.9012 |
|  | T2DM (adjusted for BMI) | 0.0151 | 0.0274 | 0.5890 |
|  | BMI | -0.0099 | 0.0190 | 0.6098 |
|  | FG | -0.0120 | 0.0081 | 0.1614 |
|  | FI | -0.0105 | 0.0075 | 0.1883 |
|  | HOMA-IR | -0.0136 | 0.0084 | 0.1302 |
|  | HOMA-β | -0.0055 | 0.0070 | 0.4481 |
| Instant coffee | T2DM | 0.0380 | 0.1111 | 0.7547 |
|  | T2DM (adjusted for BMI) | -0.0438 | 0.0739 | 0.5950 |
|  | BMI | 0.0708 | 0.0542 | 0.2826 |
|  | FG | 0.0006 | 0.0184 | 0.9746 |
|  | FI | 0.0146 | 0.0191 | 0.4994 |
|  | HOMA-IR | 0.0168 | 0.0262 | 0.5675 |
|  | HOMA-β | 0.0061 | 0.0180 | 0.7560 |
| Other type | T2DM | -0.0051 | 0.0054 | 0.3609 |
|  | T2DM (adjusted for BMI) | -0.0026 | 0.0064 | 0.6883 |
|  | BMI | -0.0075 | 0.0052 | 0.2078 |
|  | FG | 0.0068 | 0.0116 | 0.5868 |
|  | FI | 0.0131 | 0.0128 | 0.3526 |
|  | HOMA-IR | 0.0150 | 0.0147 | 0.3526 |
|  | HOMA-β | 0.0116 | 0.0072 | 0.1673 |
